# Hemoglobin Adsorption/Desorption Studies of New Generation Stimuli Responsive Polyacrylic Acid‐Cu^2+^ Hybrid Nanoflowers (PAA@Cu^2+^ hNFs)

**DOI:** 10.1002/cbdv.71762

**Published:** 2026-09-27

**Authors:** Merve Durmus, Nalan Özdemir

**Affiliations:** ^1^ Spectroscopy@IKU, IKU‐SPECTRA Molecular Sciences and Spectroscopy Applied Research Center, Istanbul Kultur University Istanbul Türkiye; ^2^ Department of Common Courses Rectorate, Istanbul Kultur University Istanbul Türkiye; ^3^ Chemistry, Graduate School of Natural and Applied Sciences, Erciyes University Kayseri Türkiye; ^4^ Department of Chemistry, Faculty of Science Erciyes University Kayseri Türkiye

**Keywords:** metal‐organic hybrid nanoflowers, protein separation, stimuli‐responsive polymers

## Abstract

In our study, pH‐responsive poly(acrylic acid)‐Cu^2+^ hybrid nanoflowers (PAA@Cu^2+^ hNFs) were fabricated by using poly(acrylic acid) and Cu^2+^ ions in phosphate‐buffered saline solution (PBS). For comparison, pure copper phosphate nanoflowers (Cu NFs) were fabricated. Synthesized PAA@Cu^2+^ hNFs and Cu NFs were characterized using SEM, EDX, XRD, FTIR, BET, zeta potential, and DLS measurements. Within the pH range of 6–9, PAA@Cu^2^^+^ hNFs exhibit a spherical, flower‐like porous morphology, with the pH 9 sample showing a more compact, uniform structure and more homogeneous interpetal pores than those at other pH values. Then, hemoglobin (Hb) adsorption studies were also performed using PAA@Cu^2+^ hNFs and Cu NFs across various parameters, including pH, Hb concentration, ionic strength, temperature, and time. At pH 7, the highest Hb adsorption capacities of PAA@Cu^2+^ hNFs and Cu NFs were calculated as 961.98 and 94.23 mg/g, respectively. The desorption studies of Hb from the synthesized nanoflowers were performed in an acetate buffer solution including NaCl. The desorption yields of Hb for PAA@Cu^2+^ hNFs and Cu NFs were calculated as 96.1% and 95.8%, respectively. Then, the reusability of the synthesized nanoflowers for Hb adsorption was evaluated over five cycles. These results suggest that PAA@Cu^2^^+^ hNFs are suitable for Hb separation.

## Graphical abstract

A new pH‐responsive poly(acrylic acid)‐Cu^2^^+^ hybrid nanoflower is developed for efficient hemoglobin adsorption. The material combines a porous flower‐like architecture with stimuli‐responsive behavior, achieving high adsorption capacity, efficient desorption, and reusability. These properties demonstrate its potential as a promising adsorbent for protein adsorption and bioseparation.

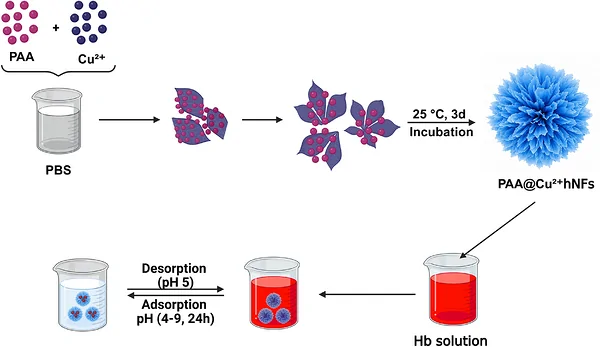

## 1 Introduction

Organic‐inorganic hybrid nanoflowers (OI‐hNFs) are materials with flower‐like structures composed of both organic and inorganic components, exhibiting properties of both components [1, 2, 3]. As a class of new‐generation materials, OI‐hNFs are of great interest due to the advantages of the strong synergistic effect that occurs from the interactions between the inorganic and organic components in their structure [4, 5]. OI‐hNFs that are formed by nucleation, growth, and completion steps are micro‐sized flower‐shaped hybrid structures that have nano‐sized leaves [6]. They are formed by binding an organic component to a metal ion, and in this synthesis, strong coordination bond interactions play a remarkable role [7, 8]. OI‐hNFs have several superior characteristics including a high surface‐to‐volume ratio, high adsorption capacity due to high porosity, high stability, sensitivity, mechanical strength, non‐toxicity, selectivity, enhanced activity, easy synthesis, low cost, and reusability [9, 10]. Due to these superior properties, OI‐hNFs have been shown to be useful in many applications, such as catalytic systems [11], drug delivery [12], enzyme mimetics [13], and tissue engineering [14]. In addition to these application area studies, several studies have demonstrated that these materials exhibit strong adsorbent properties [10, 15, 16]. In recent years, several studies have been carried out on the adsorbent properties of OI‐hNFs for applications such as dye adsorption [17], heavy metal removal [18], and protein separation [19]. However, these studies are quite limited, especially compared with those conducted using traditional adsorbent materials for protein separation.

Over the last quarter century, stimuli‐responsive polymeric materials have been synthesized by drawing inspiration from macromolecules such as proteins, polysaccharides, and nucleic acids with the development of materials science and technologies [20]. Stimulus‐responsive polymers have the ability to adapt reversibly or irreversibly to changing environmental conditions such as pH, temperature, light, ions, electrical fields, concentration, and mechanical stress by changing their chemical structure, conformation, or physical properties, thanks to the presence of functional groups that can interact with each other in their structures [21]. These materials are utilized in various fields such as medicine, food, textiles, pharmacy, chemistry, and biomedicine [22, 23, 24, 25, 26, 27]. Over the last decade, stimuli‐responsive polymeric materials have made the most important contribution to the development of organic‐metal hybrid materials toward a key technology and transferred them to industry [28].

Proteins participate in critical processes, including enzymatic, hormonal, structural, and defensive functions in organisms. Therefore, the separation of proteins from their environment is very important for determining their functions and explaining their biochemical reactions, especially for the industrial use of catalytic proteins (enzymes) [29]. In protein separation processes, various traditional methods and materials, such as aqueous two‐phase extraction, solid‐phase extraction, isoelectric precipitation method, organic solvent precipitation method, dialysis, ultrafiltration, chromatography, electrophoresis, molecular imprinting, microfluidic chips, magnetic separation, reverse micelles, and crystallization [30] are used. Although these traditional methods and materials are used extensively, they have several disadvantages, such as being expensive, requiring complex preparation processes, and requiring expert personnel. New materials are currently being developed to address these disadvantages. One of these new materials is OI‐hNFs used as supporting materials [16]. OI‐hNFs have strong selectivity for proteins through interactions such as electrostatic interactions, hydrogen bonds, and hydrophobic interactions [16, 31].

Poly(acrylic acid) (PAA) is a nontoxic, biocompatible, biodegradable, and inexpensive weak polyelectrolyte. It is synthesized by polymerization of acrylic acid. It contains ionizable carboxylic groups. These groups, in their structure, are capable of donating or accepting protons in response to changes in environmental pH. Thus, it responds to altered environmental conditions by changing its macroscopic physical and chemical properties [32]. It has been used extensively in various applications such as drug delivery, protein bioseparation, coating, biosensors, and the removal of dyes and heavy metals nowadays [33]. PAAs are notable for their role in the design of new functional materials and have been used in numerous studies to develop innovative materials. Shao and coworkers prepared submicron‐sized Fe_3_O_4_@silica core/shell microspheres by the sol‐gel method. Then, they modified PAA with these microspheres. Finally, they used this new material they synthesized (Fe_3_O_4_@silica‐g‐PAA) in the separation of lysozyme [34]. Chen et al. synthesized a pH‐responsive poly(N‐isopropylacryamide‐co‐acrylic acid) hydrogel by an aqueous dispersion polymerization process. This hydrogel was used to isolate hemoglobin from human whole blood [35]. In another study, Müller and coworkers synthesized papain/PAA nanoparticles through the complexation of papain and PAA in solution. They examined the usability of these nanoparticles for oral drug delivery [36]. Mahdavian et al. first synthesized superparamagnetic nanoparticles modified subsequently with 3‐aminopropyl triethoxysilane and acryloyl chloride. These superparamagnetic nanoparticles were then grafted with acrylic acid. These superparamagnetic nanoparticles grafted with acrylic acid were used for the separation of metal ions such as Ni^2+^, Cd^2+^, Cu^2+^, and Pb^2+ ^by Mahdavian et al. [37]. Tran et al. synthesized ZIF8‐derived nanostructures coated with a (PAA) layer. After synthesis, the use of these nanostructures for the release of doxorubicin was studied [38]. As the examples show, PAA has been used across a wide range of applications to take advantage of its properties.

Hemoglobin (Hb) is a tetrameric protein composed of two α‐like and two β‐like globin chains, each bound to a heme group. The heme group, an iron‐porphyrin complex, functions as the active center and binds oxygen molecules. Hemoglobin has a molecular weight of 64 kDa. Blood and blood products are utilized in diverse clinical scenarios, including bleeding, anemia, hereditary hemoglobin disorders, trauma, emergencies, disaster and accident conditions, and surgical operations. However, insufficient global blood donation results in unmet demand for blood and blood products during emergencies. Consequently, research on the development of hemoglobin‐based oxygen carriers as alternatives to donated blood has intensified. Purification of hemoglobin is a critical step in the synthesis of these oxygen carriers. Recently, several studies have been conducted to develop effective, selective, inexpensive, and easy methods for the separation of Hb in the biomedical field [39, 40].

In our study, we synthesized a novel hybrid nanoflower and evaluated its usability for protein adsorption, particularly for hemoglobin (Hb). In our research, for the first time, PAA@Cu^2+^ hNFs hybrid nanoflowers (PAA@Cu^2+^ hNFs) were synthesized, and the usability of this material was investigated in protein adsorption, particularly for hemoglobin (Hb). For comparison, pure copper phosphate nanoflowers (Cu NFs (Cu_3_(PO_4_)_2_·3H_2_O NFs)) were also synthesized. Then, PAA@Cu^2+^ hNFs and pure Cu NFs (Cu_3_(PO_4_)_2_·3H_2_O NFs) were characterized, and their usability as adsorbents for Hb separation was investigated. Specifically, the effects of parameters such as pH, Hb concentration, ionic effect, temperature, and time on Hb adsorption were investigated. Hb was chosen as the model protein to meet the need for blood and blood products. To characterize the synthesized PAA@Cu^2+^ hNFs and Cu_3_(PO_4_)_2_·3H_2_O NFs, some studies were performed. First, the morphologies of PAA@Cu^2+^ hNFs and Cu_3_(PO_4_)_2_·3H_2_O NFs were evaluated using scanning electron microscopy (SEM). Zeta potential, energy‐dispersive x‐ray (EDX), dynamic light scattering (DLS), x‐ray diffraction (XRD), and Fourier transform infrared spectroscopy (FTIR) analyses were also performed.

To the best of our knowledge, the synthesis and application of PAA@Cu^2+^ hNFs for Hb separation is the first report in the literature. The high adsorption capacity, caused by the large surface area, the huge porous space between petals, and the surface charge on the PAA@Cu^2+^ hNFs, played a crucial role in the protein separation. The data obtained from the study indicate that the PAA@Cu^2+^ hNFs we synthesized and characterized have potential for use in the separation of Hb.

## 2 Materials and Methods

### 2.1 Chemicals

Bovine hemoglobin (Hb; Sigma‐Aldrich, CAS 9008‐02‐0, product no. H2625; prepared from washed, lysed, and dialyzed bovine erythrocytes; predominantly in the ferric methemoglobin form, Fe^3^^+^), bovine serum albumin (BSA), copper(II) sulphate pentahydrate (CuSO_4_·5H_2_O), sodium dihydrogen phosphate (NaH_2_PO_4_), acrylic acid, ammonium persulfate (APS), sodium chloride (NaCl), sodium hydroxide (NaOH), and potassium chloride (KCl) were purchased from Sigma‐Aldrich (USA). Potassium phosphate dibasic (KH_2_PO_4_), hydrochloric acid (HCl), polydimethylsiloxane (PDS), acetic acid, sodium acetate, and disodium hydrogen phosphate (Na_2_HPO_4_), were obtained from Merck. Commassie plus protein (Bradford) assay reagent was purchased from Thermo Scientific, USA. Analytical‐grade reagents were used, and all experimental solutions were prepared with ultrapure water.

### 2.2 Synthesis of PAA

PAA was synthesized by free‐ radical polymerization of acrylic acid monomers [41]. First, 14.9 g of acrylic acid was added to 26 mL of distilled water containing 0.2 g of NaOH, and the mixture was stirred magnetically at 25°C. Then, separately, 0.35 g of APS and 0.34 g of PDS, dissolved in 3 mL of distilled water, were added to the first prepared monomer solution. For polymerization, this mixture was kept in a water bath at approximately 60°C. The PAA synthesis was studied at pH 4 value. As the polymer forms, the viscosity of the reaction mixture increases visibly.

### 2.3 Synthesis of PAA@Cu^2+^ hNFs and Pure Cu NFs

The synthesis procedure for the PAA@Cu^2+^ hNFs used in our study is quite similar to that used for the hybrid nanoflowers synthesized in our previous studies [40, 42]. For the synthesis of PAA@Cu^2+^ hNFs, a 120 mM Cu^2+^ ion stock solution was first prepared. A certain amount of the Cu^2+^ ion stock solution was added to phosphate‐buffered saline (PBS) solution (10 mM, pH 5–9) containing 0.02 mg/mL PAA to achieve a final Cu^2+^ ion concentration of 0.8 mM. Then this mixture was robustly vortexed for 30 s and incubated at room temperature for 3 days. After this time, the obtained blue precipitates were centrifuged at 6000 rpm for 10 min and washed three times with pure water to remove free components. Washed precipitates were dried at 30°C.

Pure copper phosphate nanoflowers (Cu NFs) were synthesized to demonstrate the effectiveness of PAA for the adsorption of Hb. For the Cu NFs synthesis, a certain amount of the stock Cu^2+^ ion solution was added to a PAA‐free PBS solution (10 mM, pH 5–9) to achieve a final concentration of 0.8 mM Cu^2+16^. The subsequent steps of the synthesis were carried out in the same manner as described above.

### 2.4 Characterization of the PAA@Cu^2+^ hNFs and Pure Cu NFs

The morphology of the PAA@Cu^2+^ hNFs was obtained by scanning electron microscopy (SEM; ZEISS EVO LS10). Energy‐dispersive x‐ray (EDX; ZEISS EVO LS10) analysis was used to investigate the elemental compositions of the PAA@Cu^2+^ hNFs. Fourier transform infrared spectrophotometer (FTIR; PerkinElmer 400) and x‐ray diffraction (XRD; Bruker AXS D8) were used to illuminate the chemical structure and the crystal structure of the PAA@Cu^2+^ hNFs. Zeta potential measurements and dynamic light scattering (DLS) of the PAA@Cu^2+^ hNFs were measured by Litesizer500. The pore size, total pore volume, and surface area of ​​ PAA@Cu^2+^ hNFs were determined by BET analysis. All analyses and measurements made for PAA@Cu^2+^ hNFs were also carried out for pure Cu NFs.

### 2.5 Hb Adsorption Studies of PAA@Cu^2+^ hNFs and Pure Cu NFs

The adsorption capacity of PAA@Cu^2+^ hNFs was evaluated for the separation process of Hb. A water‐bath shaker (Nüve ST 402) was used for all Hb adsorption experiments on PAA@Cu^2+^ hNFs. To determine the adsorption capacity of PAA@Cu^2+^ hNFs, Hb was dissolved in phosphate buffer solution (0.02 M) at a concentration of 1 mg/mL. Then 3 mg of hNFs was added to 3 mL of the Hb solution. This mixture was incubated for 1 day. After incubation, the mixture was centrifuged at 5000 rpm for 5 min, and the amount of adsorbed Hb was found by measuring the remaining Hb in the supernatant. Hb concentrations in the supernatants were determined using the Bradford method [43] and calculated from a calibration curve derived from these results (Figure S1). The effects of some parameters, such as pH (pH 4–9), initial Hb concentration (0–2 mg/mL), NaCl concentration (0–1 M), and temperature (+4°C–35°C), on the adsorption capacity of PAA@Cu^2+^ hNFs were studied. All adsorption studies conducted for PAA@Cu^2+^ hNFs were also performed for pure Cu NFs, and the results obtained for PAA@Cu^2+^ hNFs and pure Cu NFs were compared.

The adsorption capacity (Q) of PAA@Cu^2+^ hNFs or pure Cu NFs was calculated by Equation (1)

(1) $$Q=\frac{\left(Co-Cs\right)V}{m}$$

Co and Cs; Initial and final Hb concentrations in the supernatants, respectively (mg/mL)

V: Solution volume (mL)

m: Quantity of PAA@Cu^2+^ hNFs or pure Cu NFs (mg)

### 2.6 Hb Desorption Studies of PAA@Cu^2+^ hNFs and Pure Cu NFs

A water‐bath shaker (Nüve ST 402) was used for all Hb desorption experiments from PAA@Cu^2+^ hNFs. To evaluate the desorption yield of Hb from PAA@Cu^2+^ hNFs, Hb‐adsorbed PAA@Cu^2+^ hNFs were added to a 0.025 M acetic acid buffer with 1 M NaCl (at pH 5). Then, this mixture was incubated for 1 day. After incubation, the mixture was centrifuged at 5000 rpm for 5 min, and the amount of Hb released was determined by measuring the absorbance of the supernatant using the Bradford method. Concentrations were analyzed using the Bradford method and calculated by generating a calibration curve from the results. All desorption studies conducted for PAA@Cu^2+^ hNFs were also carried out for pure Cu NFs, and the results obtained for PAA@Cu^2+^ hNFs and pure hNFs were compared.

The desorption yield (D%) of PAA@Cu^2+^ hNFs or pure Cu NFs was calculated by Equation (2)

(2) $$D\%=\left(\frac{Cd}{Ca}\right)\times 100$$

*Ca*: Concentration of Hb adsorbed to PAA@Cu^2+^ hNFs or pure Cu NFs under optimum conditions (mg/mL)

*Cd*: After the incubation, the remaining Hb concentration in the supernatant (mg/mL)

### 2.7 Reusability of PAA@Cu^2+^ hNFs and Pure Cu NFs

According to these adsorption and desorption results, the optimum conditions were determined. Reusability of PAA@Cu^2+^ hNFs and pure Cu NFs was evaluated for five cycles using the same procedure.

### 2.8 Statistical Analysis

All experiments were independently performed in triplicate (*n* = 3). Experimental results are presented as mean ± standard deviation (SD). No data transformation, normalization, or outlier exclusion was applied prior to data analysis. Standard deviations were calculated using Microsoft Excel (Microsoft Corporation, Redmond, WA, USA). Error bars shown in the figures represent the standard deviation of three independent experiments.

## 3 Results and Discussion

**FIGURE 1:**
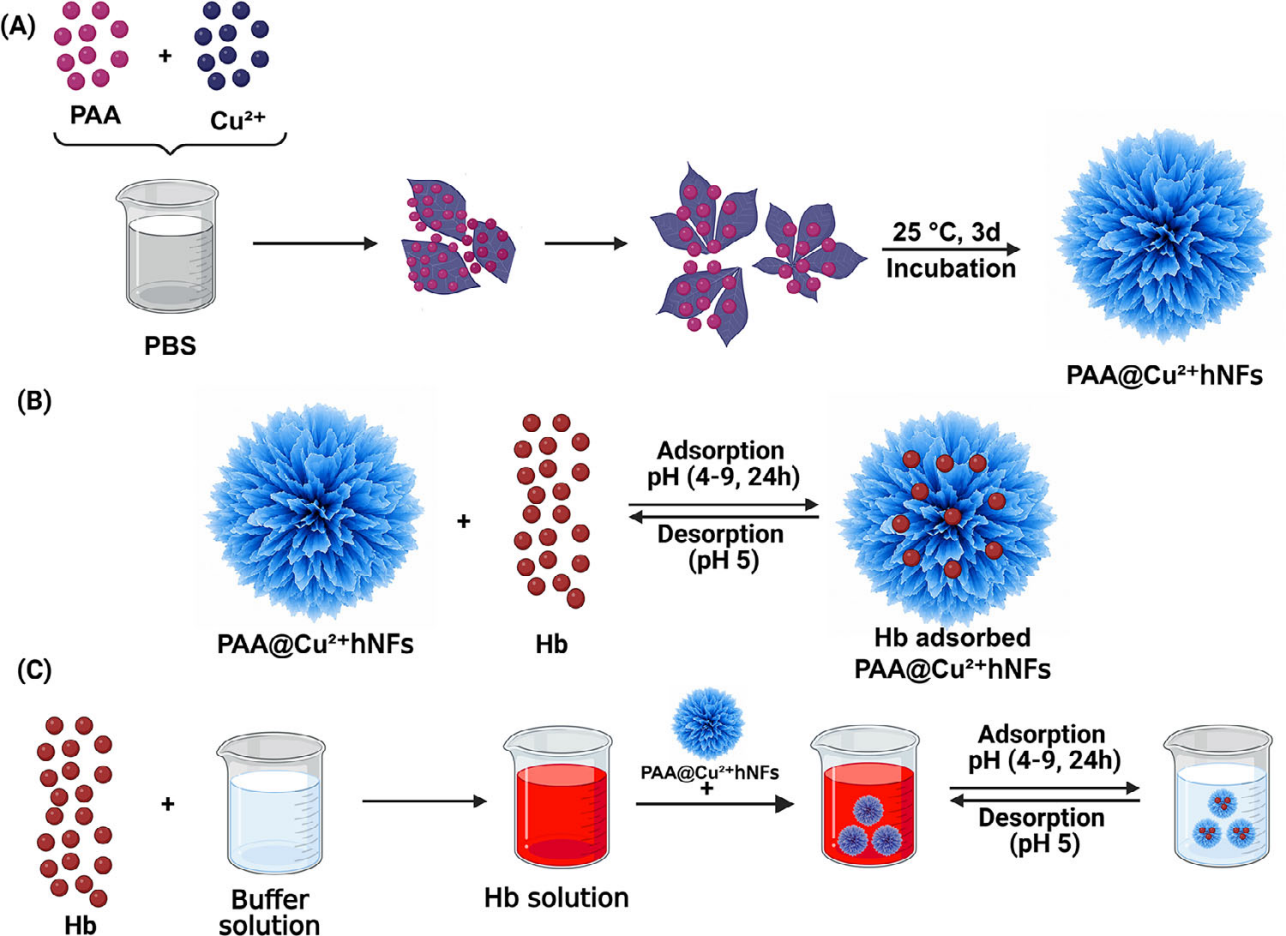
(A) The schematic diagram of the synthesis process of PAA@Cu^2+^ hNFs, (B) The diagram of the reversible adsorption‐desorption process of Hb onto PAA@Cu^2+^ hNFs, (C) The schematic diagram of the Hb separation by PAA@Cu^2+^ hNFs.

Although various traditional methods have been used for protein separation, we propose a new material with high adsorption capacity, ease of synthesis, low cost, and reusability. The synthesis of PAA@Cu^2+^ hNFs, and their use for the separation of Hb were simply illustrated in Figure 1. As shown in Figure 1, first, PAA@Cu^2+^ hNFs were fabricated by reacting PAA with Cu^2+^ ions in a 10 mM PBS solution. Then, synthesized PAA@Cu^2+^ hNFs were used for Hb separation, and it was found that they exhibited a high adsorption capacity for Hb. On the other hand, pure Cu NFs were synthesized as a control, and the same conditions and procedures were applied for Hb separation.

### 3.1 Synthesis and Characterization of PAA@Cu^2+^ hNFs and Pure Cu NFs

PAA@Cu^2+^ hNFs and pure Cu NFs were synthesized. PAA is a weak polyelectrolyte with ionizable carboxylic side‐chain groups that can donate or accept protons in response to pH variations, enabling it to adjust its macromolecular conformation according to environmental changes. Therefore, the PAA thus serves significant roles in the development of novel functional stimuli‐sensitive materials. The synthesis of PAA@Cu^2+^ hNFs, using PAA and Cu^2+^ ions as the organic and inorganic components, is the first reported in the literature. The synthesis of PAA@Cu^2+^ hNFs is a pretty straightforward process based on the coordination between PAA and Cu^2+^ ions. The fabrication process of PAA@Cu^2+^ hNFs consists of three steps. In the first step (nucleation), Cu^2+^ ions form primary Cu_3_(PO_4_)_2_·3H_2_O nanocrystals with phosphate anions in the PBS solution. These Cu_3_(PO_4_)_2_·3H_2_O crystals serve as nucleation sites for the formation of nanopetals. In the second step (growth), PAA binds to Cu_3_(PO_4_)_2_·3H_2_O crystals, forming large agglomerates that form the nanopetals during incubation. These agglomerates grow anisotropically and continuously. In the third step (completion), flower‐like structures called nanoflowers are formed from large agglomerates that formed the nanopetals as a result of anisotropic growth [44]. In brief, Cu^2^^+^ ions coordinate with PAA through carboxyl groups, while simultaneously forming the copper phosphate complex Cu_3_(PO_4_)_2_·3H_2_O as Cu^2+^ ions bind to phosphate ((PO_4_)^3−^) groups. This process drives the growth and formation of nanopetals, which subsequently self‐assemble into hybrid nanoflowers.

The synthesized PAA@Cu^2+^ hNFs and pure Cu NFs were systematically characterized using SEM, EDX, XRD, FTIR, BET, zeta potential, and DLS measurements. PAA@Cu^2+^ hNFs were fabricated in the PBS pH value range from 5 to 9, at room temperature for 3 days. There was no product formed at pH 5 because of the strong repulsion between PAA and Cu^2+^ ions.

**FIGURE 2:**
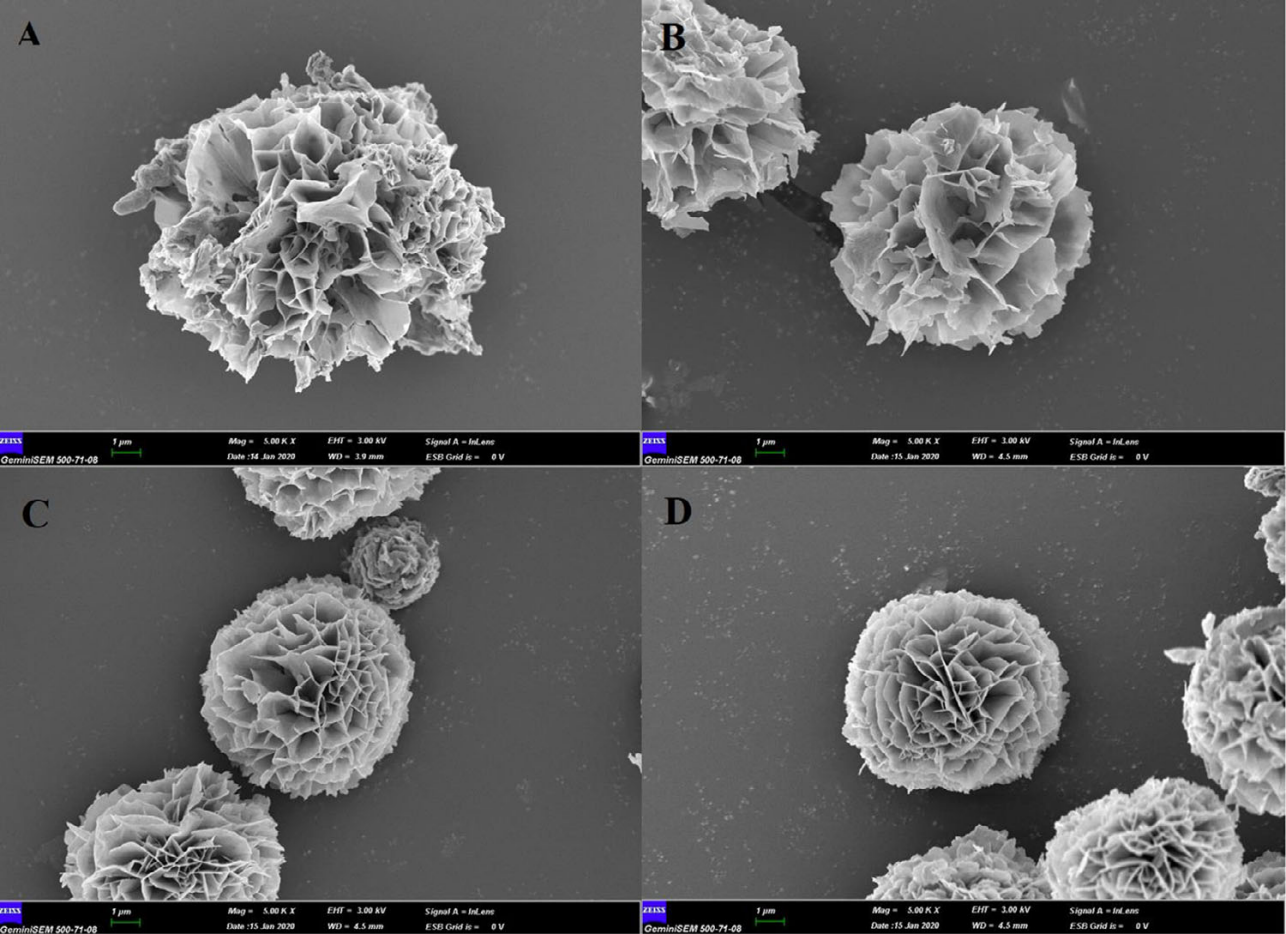
SEM images of the PAA@Cu^2+^ hNFs synthesized at different pH levels at room temperature (A) pH 6, (B) pH 7, (C) pH 8, and (D) pH 9.

The morphology of PAA@Cu^2+^ hNFs at pH 6, 7, 8, and 9 was characterized via SEM. SEM images of the synthesized PAA@Cu^2+^ hNFs at different pH levels are given in Figure 2. As shown in Figure 2, all the synthesized PAA@Cu^2+^ hNFs are spherical, highly porous, have large surface areas, and are flower‐like. The diameters of all PAA@Cu^2+^ hNFs were determined by averaging and measuring from SEM images with the help of the ImageJ program. The average size of hNFs that were synthesized at pH 6–9 is given in Figure S2. According to these measurement results, the average diameters of PAA@Cu^2+^ hNFs synthesized at pH 6, 7, 8, and 9 were 10.83 ± 0.64, 8.66 ± 1.53, 9.09 ± 1.53, and 9.14 ± 1.39 µm, respectively. When the results at pH 7, 8, and 9 are examined, the diameter increases as pH increases. Especially, the morphology of PAA@Cu^2+^ hNFs at pH 9 is much more uniform and compact compared to PAA@Cu^2+^ hNFs morphologies at other pHs. It is much more homogeneous in the areas between the petals. It is also clearly seen from Figure 2D. This high surface‐to‐volume ratio also allows for immobilization of proteins. Thanks to these unique properties, interest in flower‐like structures is growing steadily.

**FIGURE 3:**
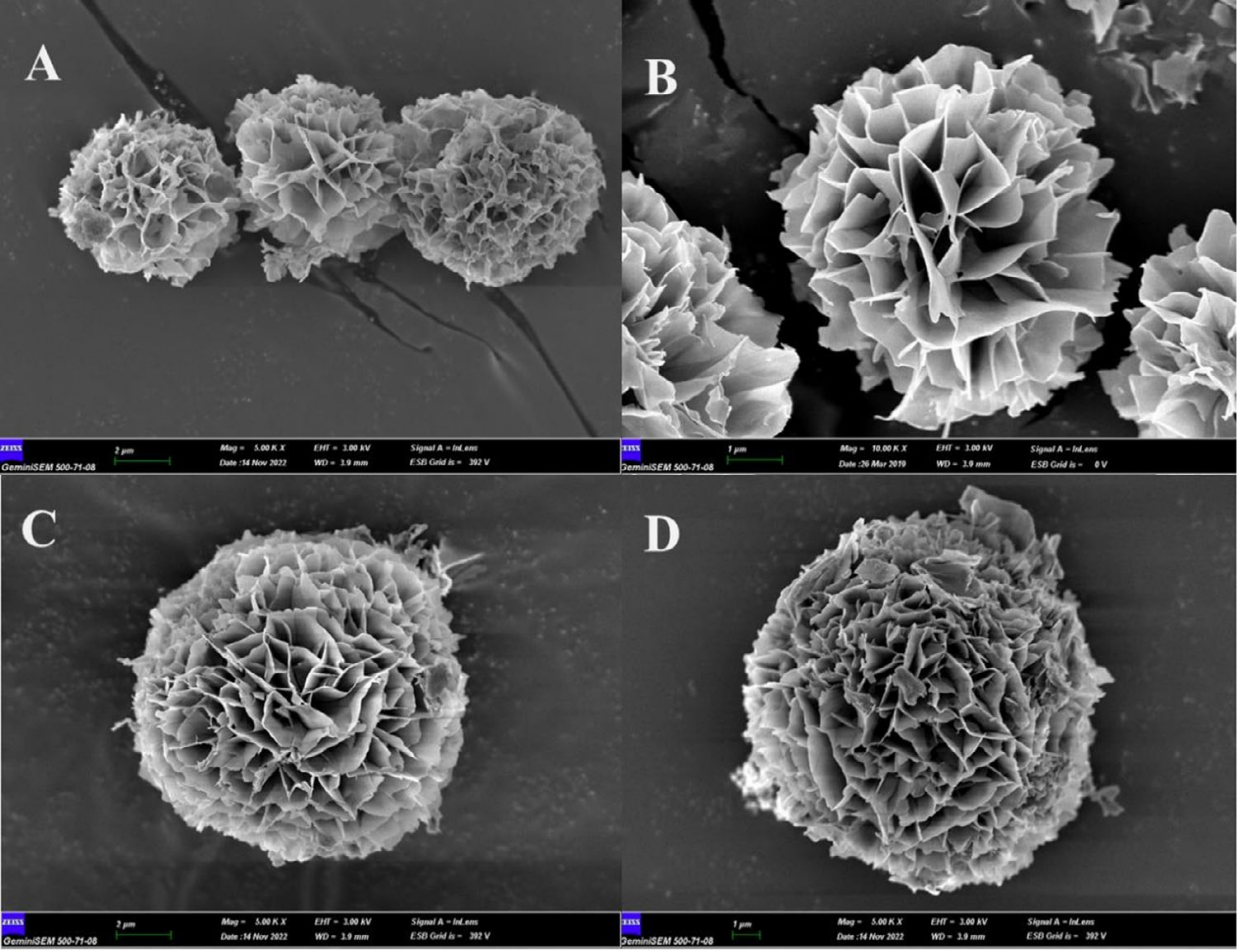
SEM images of the pure Cu NFs synthesized at different pH levels at room temperature (A) pH 6, (B) pH 7, (C) pH 8, and (D) pH 9.

SEM images of synthesized pure Cu NFs at different pH levels are given in Figure 3. The diameters of all pure Cu NFs were determined by averaging and measuring from SEM images with the help of the ImageJ program. The average size of pure Cu NFs that were synthesized at pH 6–9 (Figure S3). According to these measurement results, the average diameters of pure Cu NFs synthesized at pH 6, 7, 8, and 9 were 7.09 ± 0,82, 8.37 ± 0.92, 10.98 ± 2.64, and 12.75 ± 2.06 µm, respectively. When the results at pH 7, 8, and 9 are examined, it is seen that the diameter increases as the pH increases. As shown in Figure 3, the diameter of the flower‐shaped Cu_3_(PO_4_)_2_·3H_2_O increased with increasing synthesis pH. Önal et al. reported the synthesis and characterization of pure Cu NFs for an application different from our study. They used similar methods for the preparation and characterization of their material [31].

The pore size, total pore volume, and surface area of synthesized PAA@Cu^2+^ hNFs were determined by BET analysis. The surface area and pore size of PAA@Cu^2+^ hNFs synthesized at pH 9 were 115.14 m^2^/g and 19.30 nm, respectively. The surface area and pore size of PAA@Cu^2+^ hNFs synthesized at pH 8 were 82.26 m^2^/g and 21.92 nm, respectively. The surface area and pore size of PAA@Cu^2+^ hNFs synthesized at pH 7 were 71.14 m^2^/g and 29.82 nm, respectively (Table S1). According to SEM images, surface areas, and pore sizes of PAA@Cu^2+^ hNFs, pH 9 was determined to be the optimum synthesis pH for PAA@Cu^2+^ hNFs. For this reason, subsequent studies were carried out with PAA@Cu^2+^ hNFs synthesized at pH 9. Similarly, the same analyses were performed for Cu NFs. The surface area and pore size of Cu NFs, synthesized at pH 9, were found to be 44.27 m^2^/g and 15.72 nm, respectively (Table S1).

**FIGURE 4:**
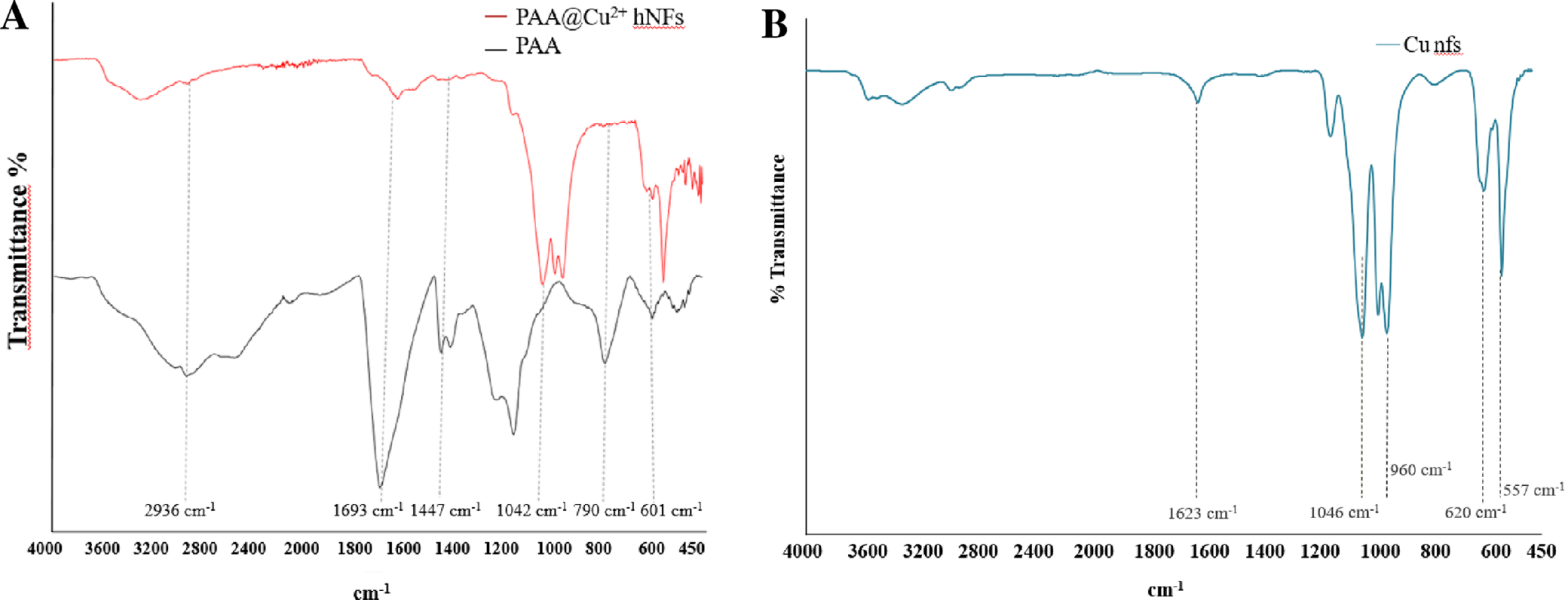
FTIR spectra of (A) PAA@Cu^2+^ hNFs and PAA, (B) pure Cu NFs.

To investigate the chemical and crystal structures and the elemental composition of the PAA@Cu^2+^ hNFs, FTIR, XRD, and EDX analyses were performed. The chemical structure and interaction between the components of PAA@Cu^2+^ hNFs were explained by FTIR. As shown in Figure 4A, the typical absorption peaks of PAA@Cu^2+^ hNFs and PAA were observed. The characteristic peaks at 2936, 1693, 1447, and 1419 cm^−1^ of PAA were assigned to ─CH_2_─ (stretching and bending), ─COO (stretching in ─COOH), and C─O (stretching in ─COOH), respectively. These results are quite similar to the results obtained by Lertsanawut et al. [45]. The peaks at ∼1640, ∼1042, and ∼998 cm^−1^ of PAA@Cu^2+^ hNFs were attributed to ─COO (stretching in ─COOH), P═O (stretching), and P─O (stretching), respectively. The presence of bending vibrations of O═P─O groups was observed as characteristic peaks at ∼601 and ∼546 cm^−1^. The peak at range ∼2930–3600 cm^−1^ indicated the existence of the ─CH_2_ group. According to these results, it can be said that PAA was successfully incorporated into the structure of PAA@Cu^2+^ hNFs. As shown in Figure 4B, the typical absorption peaks of Cu NFs are indicated. The bending vibration of O═P─O groups was the obtained characteristic peaks at ∼620 and ∼557 cm^−1^. The peaks at ∼960 and ∼1042 cm^−1^ of pure Cu NFs were attributed to P─O (stretching) and P═O (stretching), respectively. These peaks proved the formation of PAA@Cu^2+^ hNFs and pure Cu NFs.

**FIGURE 5:**
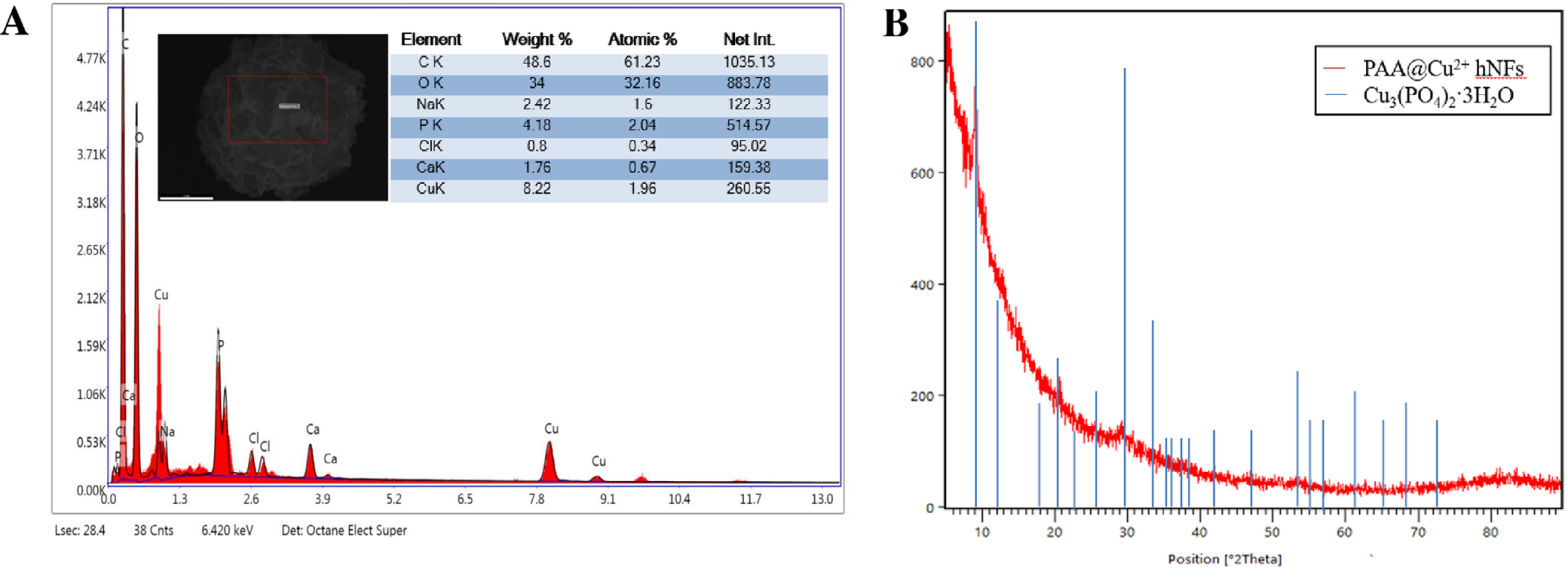
(A) EDX results of the PAA@Cu^2+^ hNFs (B) XRD patterns of PAA@Cu^2+^ hNFs. (B) The peak position of the Cu_3_(PO_4_)_2_·3H_2_O (JCPDS 00‐22‐0548) located for comparison.

The elemental composition of the synthesized PAA@Cu^2+^ hNF and Cu NFs was analyzed using EDX. They can be clearly seen in Figures 5A, S4, and S5. The elemental distribution expressed as weight and atomic percentages of Cu, O, C, and P is shown for PAA@Cu^2+^ hNFs. In particular, the elemental distribution, in terms of weight and atomic percentages of Cu, O, and P, indicates the presence of Cu_3_(PO_4_)_2_ in PAA@Cu^2+^ hNFs. The C peaks in the figure stemmed from the PAA in the structure. As these results indicate, the presence of Cu^2+^ ions in the structure is evidenced by the weight and atomic percentage in the spectrum.

The crystal structure of hNFs was examined by XRD. The crystal patterns of PAA@Cu^2+^ hNFs and pure Cu NFs were depicted in Figures 5B and S6, respectively. The peak positions and intensities of PAA@Cu^2+^ hNFs and pure Cu NFs were matched with the XRD pattern of Cu_3_(PO_4_)_2_·3H_2_O (JCPDS card: 00‐22‐0548). According to these results, the inclusion of the most diffraction peaks of Cu_3_(PO_4_)_2_·3H_2_O in the PAA@Cu^2+^ hNFs proves the presence of Cu^2+^ ions in the hNFs structure, and it is seen that Cu_3_(PO_4_)_2_·3H_2_O is effective in the crystalline structure of PAA@Cu^2+^ hNFs.

**FIGURE 6:**
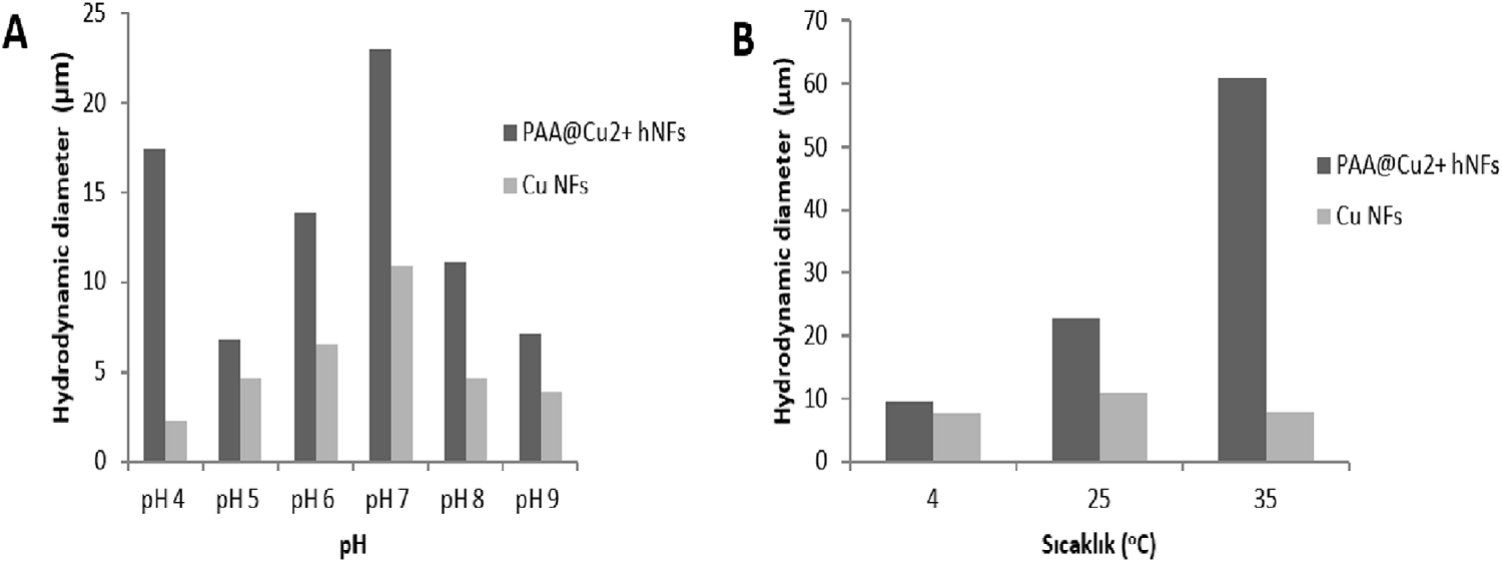
Hydrodynamic diameter changes of PAA@Cu^2+^ hNFs and pure Cu NFs at different (A) pHs, and (B) temperatures.

Hydrodynamic diameter changes of PAA@Cu^2+^ hNFs and pure Cu NFs at different pHs (4–9) and temperatures (+4°C–35°C) were identified by DLS analysis (Figure 6). As shown in Figure 6A, PAA@Cu^2^^+^ hNFs (dark gray) and Cu NFs (light gray) reach their highest hydrodynamic diameter at pH 7. The largest hydrodynamic diameters of PAA@Cu^2+^ hNFs and pure Cu NFs were 22.91 and 10.87 µm, respectively (pH 7). The DLS measurements demonstrated that the hydrodynamic diameter of PAA@Cu^2^^+^ hNFs (dark gray) varied significantly with changes in pH and temperature, whereas pure Cu NFs (light gray) exhibited much smaller changes. The smallest hydrodynamic diameter of PAA@Cu^2+^ hNFs was found to be 6.78 µm at pH 5. But, the hydrodynamic diameter of PAA@Cu^2+^ hNFs was increased again at pH 4. The smallest hydrodynamic diameter of Cu NFs was 2.26 µm at pH 4. As shown in Figure 6B, the hydrodynamic diameter of the PAA@Cu^2+^ hNFs increases with increasing temperature. However, this is not the case for Cu NFs. The largest hydrodynamic diameter of PAA@Cu^2+^ hNFs was 61.03 µm at 35°C.

To determine the isoelectric point (pI) of PAA@Cu^2+^ hNFs and pure Cu NFs, the zeta potential measurements at various pH values (pH 5–9) were performed. According to the results (Figures S7 and S8), the zeta potential values of PAA@Cu^2+^ hNFs increased dramatically as the pH decreased. The pI of PAA@Cu^2+^ NFs was reached near 0 mV at pH 4 (Figure S7). Additionally, the zeta potential of pure Cu NFs increased as the pH decreased. The zeta potential of pure Cu NFs was reached near 0 mV at a pH ∼4.5 (Figure S8).

Based on the characterization results, pH 9 was determined to be the optimum synthesis pH for PAA@Cu^2+^ hNFs. For these reasons, subsequent studies were conducted using PAA@Cu^2+^ hNFs synthesized at pH 9.

### 3.2 Effects of pH on Hb Adsorption Onto the PAA@Cu^2+^ hNFs and Pure Cu hNFs

**FIGURE 7:**
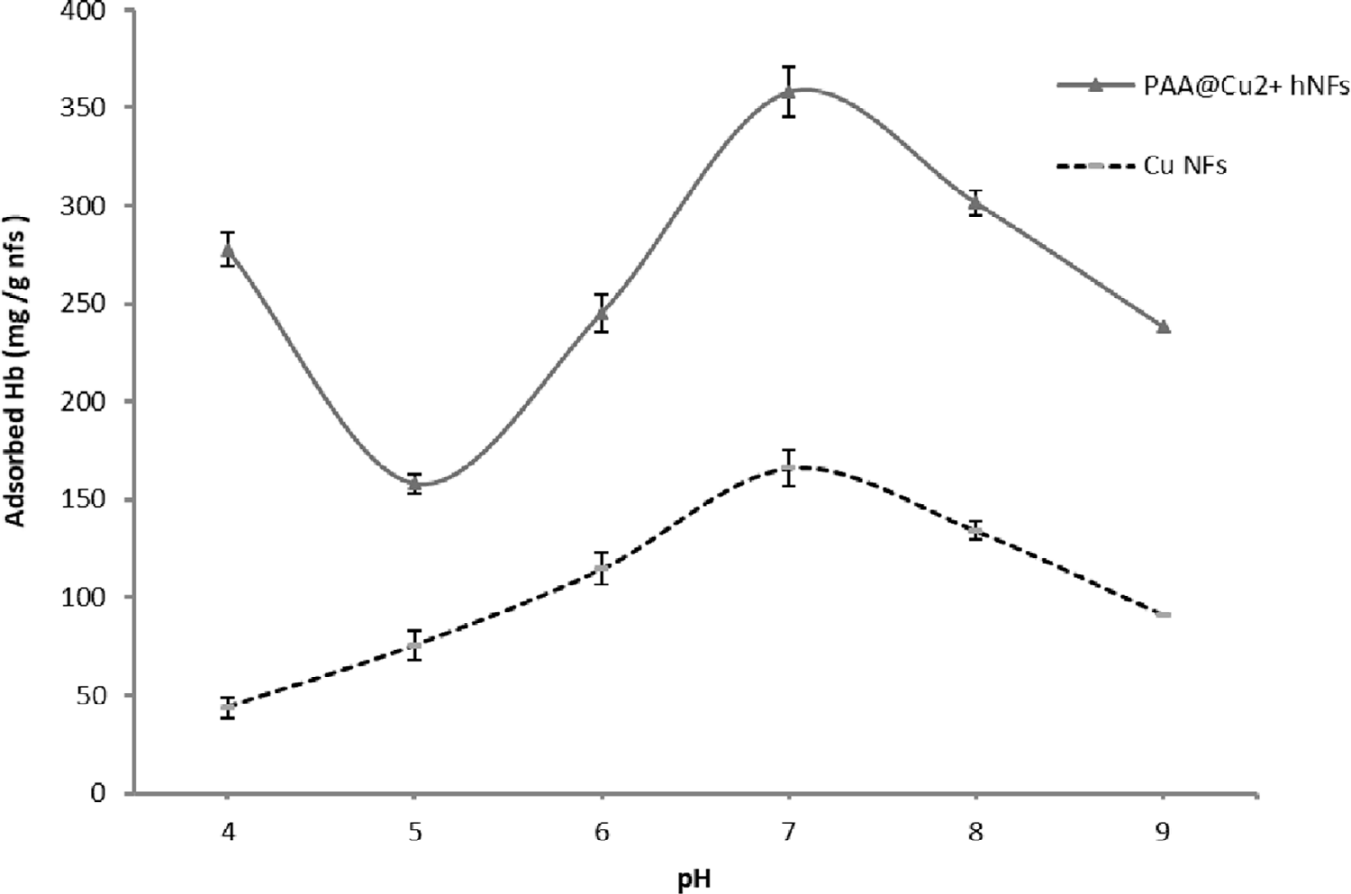
The effect of pH on the adsorption capacities of PAA@Cu^2+^ hNFs and pure Cu NFs (*n* = 3) (C_Hb_: 1 mg/mL, T: 25°C, M _phosphate buffer solution_: 0.02 M).

Various new materials have been fabricated for protein separation. Specifically, OI‐hNFs and stimuli‐responsive polymeric‐based materials are drawing attention for use in separation processes due to their specific characteristics [31, 46]. To achieve the highest adsorption capacity, it is necessary to investigate the effect of parameters such as pH, adsorbate concentration, ionic strength, and temperature. The effect of pH on Hb adsorption is given in Figure 7. As can be clearly seen in Figure 7, the effect of pH on the adsorption capacity is a crucial parameter in determining the behavior of PAA@Cu^2+^ hNFs toward Hb. Therefore, it was tested in phosphate buffer solutions ranging from pH 4 to 9 compared with pure Cu NFs. The ionization and interaction of PAA@Cu^2+^ hNFs, Cu NFs, and Hb vary with changes in pH. The highest Hb adsorption capacities of PAA@Cu^2+^ hNFs and pure Cu NFs were determined to be 358.02 and 166.52 mg/g (Figure 7), respectively. The lowest Hb adsorption capacities of PAA@Cu^2+^ hNFs and pure Cu NFs were found to be 158.11 (at pH 5) and 43.74 mg/g (at pH 4), respectively. Adsorption capacities of PAA@Cu^2+^ hNFs and pure Cu NFs were found to be different at different pHs (Figure 7).

The highest Hb adsorption capacities of both materials (PAA@Cu^2+^ hNFs and pure Cu NFs) were shown in the phosphate buffer solution (0.02 M) at pH 7. While the adsorption capacities of both materials were decreased at pH 7 above and below, the capacity of PAA@Cu^2+^ hNFs was increased again at pH 4. This is quite interesting. The observed adsorption behavior is governed by the combined effects of both the physicochemical properties and the pH‐dependent characteristics of PAA@Cu^2^^+^ hNFs and Hb. This observed adsorption behavior can be explained by two main parameters. One of them is the isoelectric points of hNFs and Hb. The other is the stimuli‐ responsive nature of PAA@Cu^2+^ hNFs. Hb and PAA@Cu^2+^ hNFs are positively charged below the isoelectric point (pI) and negatively charged above the isoelectric point (pI) [45]. Since the hemoglobin used in this study is in the ferric methemoglobin form (Fe^3^^+^), its pI is approximately 7.20, compared with 6.95 for ferrous oxyhemoglobin (Fe^2^^+^) [47, 48]. Hb is positively charged in a phosphate buffer solution below pH 7.2. However, it is negatively charged in phosphate buffer solutions at pH above 7.2. The pI of PAA@Cu^2+^ hNFs was at ∼pH 4. The structure (PAA@Cu^2+^ hNFs) is positively charged below this value, while it is negatively charged above this value. Due to the electrostatic repulsion interaction between negatively charged Hb and negatively charged PAA@Cu^2+^ hNFs above pH 7.2, the adsorption capacity of PAA@Cu^2+^ hNFs decreased [29]. Additionally, the stimuli‐sensitive nature of PAA@Cu^2+^ hNFs supports these adsorption results. The DLS measurements demonstrated that the hydrodynamic diameter of PAA@Cu^2+^ hNFs varied significantly with changes in pH and temperature, whereas pure Cu NFs exhibited much smaller changes (Figure 6). Likewise, the zeta potential measurements (Figure S6) revealed a pH‐dependent variation in the surface charge of PAA@Cu^2+^ hNFs, which originates from the protonation/deprotonation of the carboxyl groups of PAA. The carboxyl groups in PAA were ionized with a p*K*_a_ of ∼4.5. The numbers of ─COOH and a‐COO^−^ are the same at this pH, and this change in the conformation of PAA again increases the adsorption capacity of PAA@Cu^2+^ hNFs (at pH 4) [32, 49,50]. These findings indicate that the PAA‐containing hybrid nanoflowers (PAA@Cu^2+^ hNFs) undergo physicochemical changes in response to environmental stimuli (pH (Figure 6A) and temperature (Figure 6B)). Nevertheless, the adsorption behavior cannot be attributed solely to the responsiveness of the nanoflowers, since Hb also undergoes changes in surface charge around its isoelectric point. Therefore, the adsorption/desorption performance observed in this study is due to the combined contribution of the pH‐responsive nature of PAA@Cu^2+^ hNFs and the pH‐dependent characteristics of Hb.

As seen from Figure 7, the maximum Hb adsorption capacity of both materials (PAA@Cu^2+^ hNFs and pure Cu NFs) was observed at pH 7. For this reason, subsequent studies were carried out at this pH.

### 3.3 Effects of Hb Concentration on Hb Adsorption Capacities of PAA@Cu^2+^ hNFs and Pure Cu NFs

**FIGURE 8:**
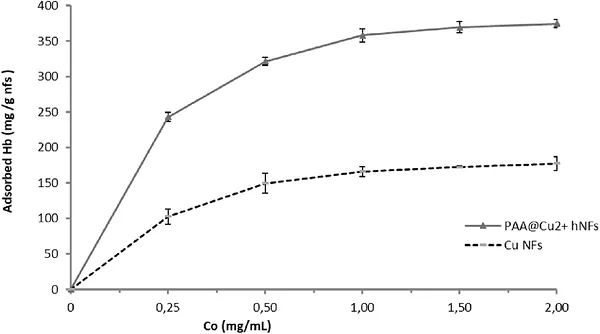
The effect of initial Hb concentrations on the adsorption capacities of PAA@Cu^2+^ hNFs and pure Cu NFs (*n* = 3) (pH 7.0, T: 25°C, M _phosphate buffer solution_: 0.02 M).

To investigate the effect of initial Hb concentration on the adsorption capacity of PAA@Cu^2+^ hNFs and pure Cu NFs, the Hb concentrations were tested from 0 to 2.00 mg/mL for both of them at pH 7. The effect of initial Hb concentrations on the adsorption capacities of PAA@Cu^2+^ hNFs and pure Cu NFs is given in Figure 8. As shown in Figure 8, the Hb adsorption capacities of PAA@Cu^2+^ hNFs and pure Cu NFs reached a plateau degree at 374.42 and 177.32 mg/g, respectively. The surfaces reached saturation after a specific capacity. The lower adsorption capacities of PAA@Cu^2+^ hNFs and pure Cu NFs were calculated to be 243.08 and 102.73 mg/g, respectively (0.25 mg/mL). At 1 mg/mL Hb concentration, the adsorption capacities of PAA@Cu^2+^ hNFs and pure Cu NFs were calculated as 358.22 and 166.37 mg/g, respectively. After this concentration, the adsorption capacity does not change significantly.

It is thought that this may be due to the multi petals, high porosity, and high surface area of the structure formed by organic and inorganic molecules [31]. Some studies have reported a strong interaction between proteins and organic/inorganic hybrid structures. These interactions are predicted to be electrostatic, hydrophobic, and coordination interactions [50, 51].

As a result, 1 mg/mL was determined to be the optimum Hb concentration of these two materials. Subsequent studies were carried out using this Hb concentration value.

### 3.4 Effects of Ionic Strength on Hb Adsorption Capacities of PAA@Cu^2+^ hNFs and Pure Cu NFs

**FIGURE 9:**
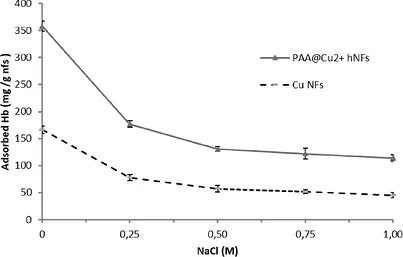
The effect of ionic strength on the adsorption capacities of PAA@Cu^2+^ hNFs and pure Cu NFs (n=3) (pH 7.0, C_Hb_: 1 mg/mL, T: 25°C, M _phosphate buffer solution_: 0.02 M).

Ionic strength plays an active role in protein adsorption. For this reason, PAA@Cu^2+^ hNFs and pure Cu NFs were tested using a buffer solution containing 0.00–1.00 M salt to see the effect of ionic strength on the adsorption of Hb to both of these materials. The effect of ionic strength on the adsorption capacities of PAA@Cu^2+^ hNFs and pure Cu NFs is given in Figure 9. As shown in Figure 9, the highest Hb adsorption capacities for both materials were observed in salt‐free buffer solutions. It is thought that there may be three different reasons for this situation. (1) The increasing ion concentration in the adsorption solution can change the surface charge of the adsorbent [52]. It can be thought that this situation changes the binding capacity of the adsorbent. (2) The solubility of the protein is decreased with the increase of salt concentrations in the solution, also known as the salting‐out effect, because of changing the hydrogen bonding capacity of water‐ Hofmeister effect [53]. (3) The conformation of proteins can be tightened with ionic strength [54]. In other words, ionic strength is an important parameter in Hb adsorption.

As a result, the buffer solution without NaCl was determined as the optimum condition.

### 3.5 Effects of Temperature on Hb Adsorption Capacities of PAA@Cu^2+^ hNFs and Pure Cu NFs

**FIGURE 10:**
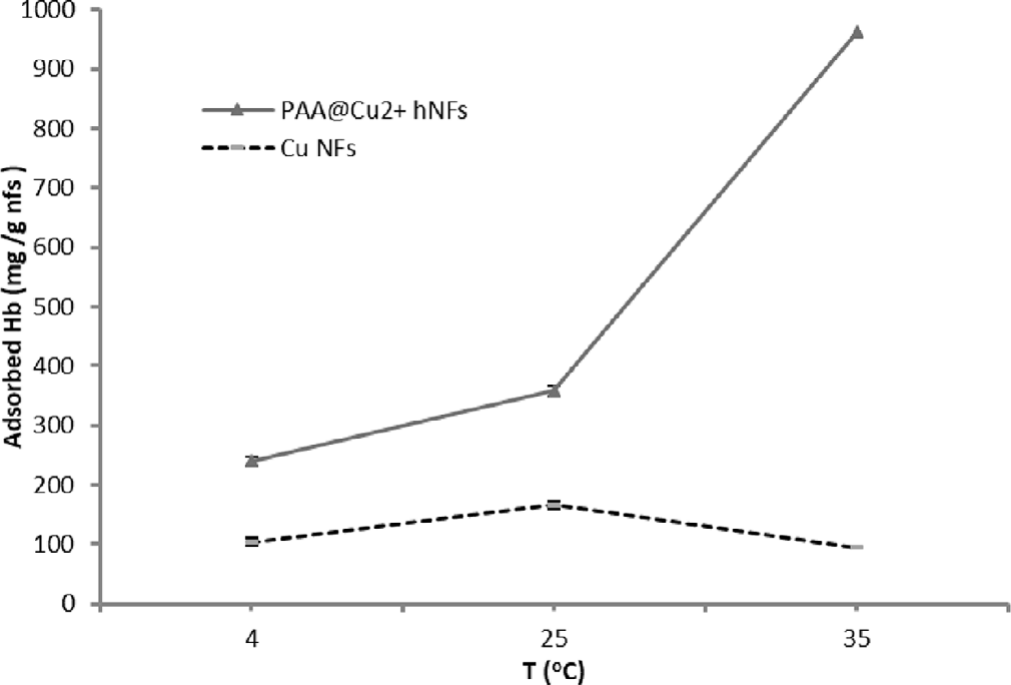
The effect of temperature on the adsorption capacities of PAA@Cu^2+^ hNFs and pure Cu NFs (*n* = 3) (pH 7.0, C_Hb_: 1 mg/mL, M _phosphate buffer solution_: 0.02 M).

It is essential to determine the effect of temperature on Hb adsorption. So, the Hb adsorption capacities of PAA@Cu^2+^ hNFs and pure Cu NFs were tested in the range of 4°C–35°C. The effect of temperature on the adsorption capacities of PAA@Cu^2+^ hNFs and pure Cu NFs is given in Figure 10. As shown in Figure 10, the highest Hb adsorption capacity for PAA@Cu^2+^ hNFs was found at 35°C, and the highest Hb adsorption capacity for pure Cu NFs was found at 25°C. At 35°C, Hb adsorption capacities of PAA@Cu^2+^ hNFs and pure Cu NFs were calculated as 961.98 and 94.23 mg/g, respectively. At 25°C, Hb adsorption capacities of PAA@Cu^2+^ hNFs and pure Cu NFs were found to be 358.02 and 166.52 mg/g, respectively. While the adsorption capacity of pure Cu NFs from 25°C to 35°C was not changed much, the adsorption capacity of PAA@Cu^2+^ hNFs was dramatically increased in the same temperature range. The determined increase in the Hb adsorption capacity of PAA@Cu^2+^ hNFs at 35°C may be due to the presence of a polymer rich in long tails in the nanoflower structure. This conformation may allow quite a lot of interaction between the carboxyl groups of PAA@Cu^2+^ hNFs and Hb [55].

### 3.6 Effect of Time on Hb Adsorption Capacities of PAA@Cu^2+^ hNFs and Pure Cu NFs

**FIGURE 11:**
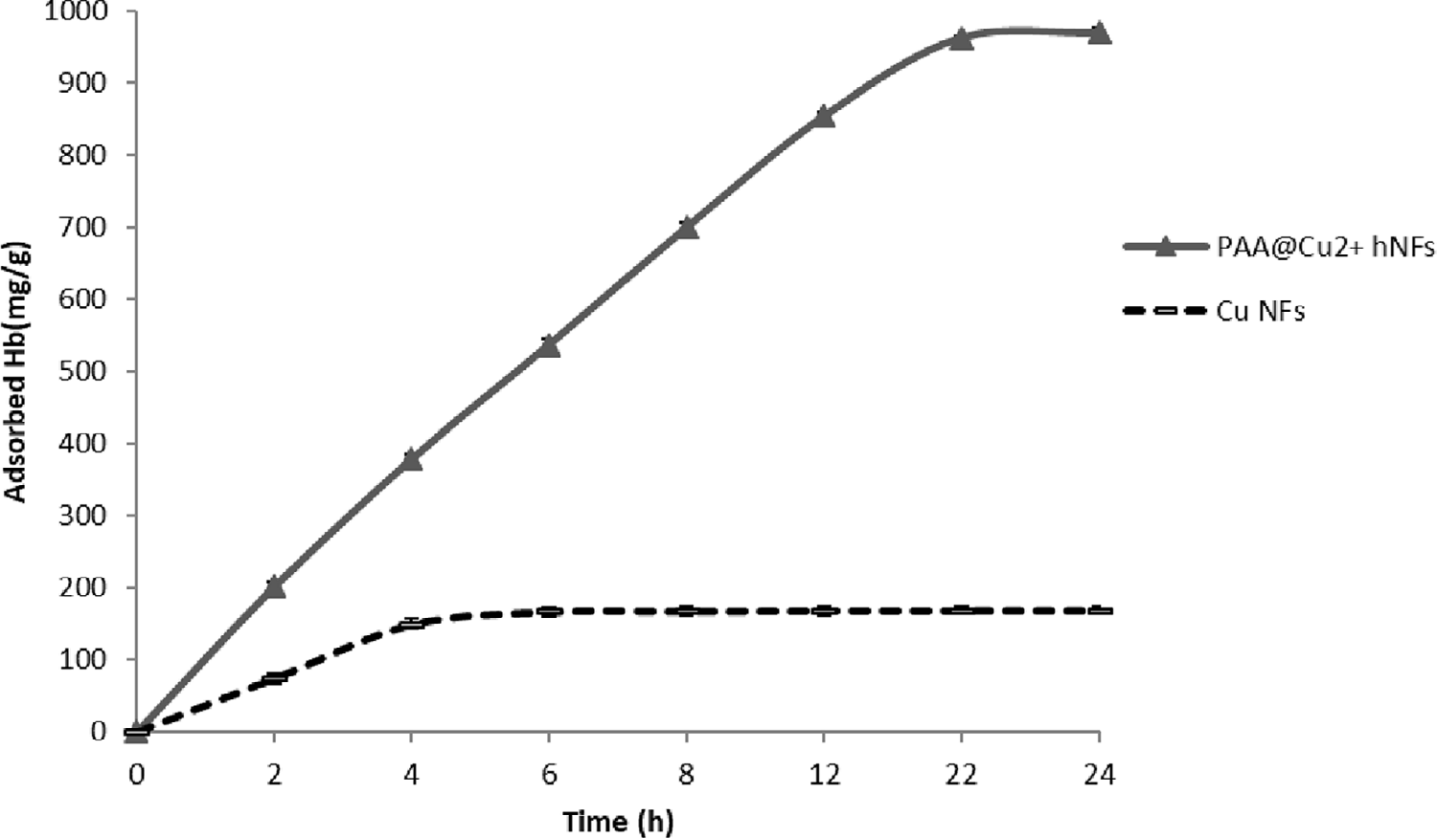
The effect of time on the adsorption capacities of PAA@Cu^2+^ hNFs and pure Cu NFs (*n* = 3) (pH 7.0, C_Hb_: 1 mg/mL, M _phosphate buffer solution_: 0.02 M).

To obtain the highest Hb adsorption capacity of PAA@Cu^2+^ hNFs and pure Cu NFs, the adsorption time was investigated over a 24‐hour period (Figure 11). At pH 7, the highest Hb adsorption capacity of PAA@Cu^2+^ hNFs was calculated as 968.79 mg/g for 24 h (C_Hb_: 1 mg/mL, T: 35°C, M _phosphate buffer solution_: 0.02 M). Hb adsorption capacity of pure PAA@Cu^2+^ hNFs was calculated as 961.78 mg/g at 22 h. Hb adsorption capacity of pure Cu_3_(PO_4_)_2_·3H_2_O NFs was calculated as 169.03 mg/g for 24 h (C_Hb_: 1 mg/mL, T: 25°C, M _phosphate buffer solution_: 0.02 M). Hb adsorption capacity of pure Cu NFs was calculated as 167.52 and 169.01 mg/g at 6 and 22 h, respectively. After 22 h, the adsorption capacity of PAA@Cu^2+^ hNFs has not changed significantly. After 6 h, the adsorption capacity of Cu NFs has not changed significantly. The total adsorption capacities of PAA@Cu^2+^ hNFs were completed for 22 h. The total adsorption capacities of Cu NFs were achieved after 6 h. While the adsorption rate is initially quite fast, it slows down after the structure reaches a certain level of saturation.

**FIGURE 12:**
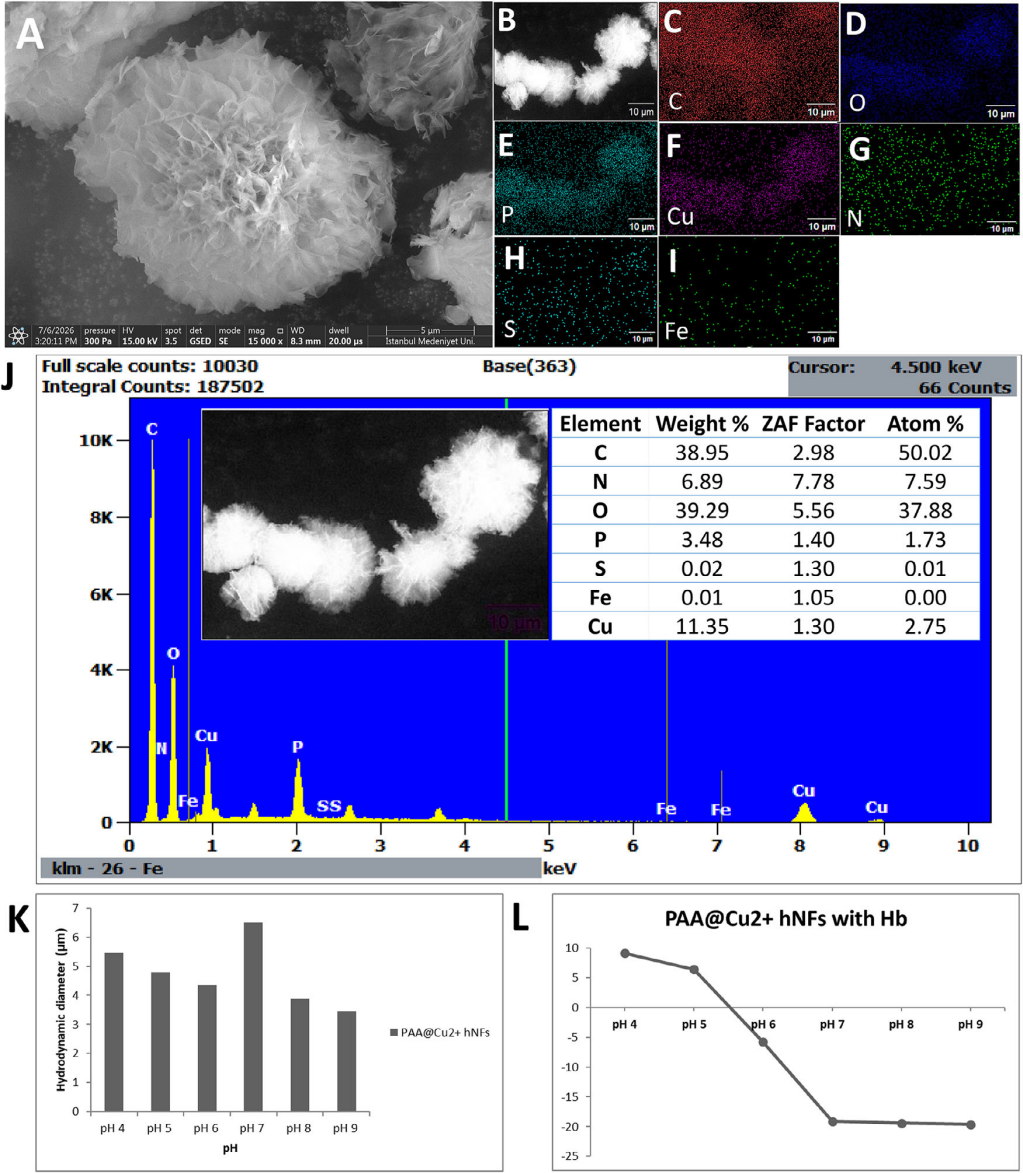
Characterization of Hb‐adsorbed PAA@Cu^2+^ hNFs. (A) SEM image showing the overall flower‐like morphology of the Hb‐loaded PAA@Cu^2+^ hNFs. (B) High‐magnification SEM image of the region selected for elemental mapping. (C–I) SEM‐EDX elemental mapping images showing the distribution of carbon (C), oxygen (O), phosphorus (P), copper (Cu), nitrogen (N), sulfur (S), and iron (Fe) across the hybrid nanoflower structure. (J) EDX spectrum and corresponding quantitative elemental composition (weight % and atomic %) of the Hb‐loaded PAA@Cu^2+^ hNFs; inset shows the mapped region. (K) Hydrodynamic diameter of Hb‐loaded PAA@Cu^2+^ hNFs at different pH values, measured by dynamic light scattering (DLS). (L) Zeta potential of Hb‐loaded PAA@Cu^2+^ hNFs measured at different pH values.

To gain further insight into the adsorption mechanism, the Hb‐loaded PAA@Cu^2+^ hNFs were further characterized and compared with the PAA@Cu^2+^ hNFs (Figure 12). SEM images (Figure 12A,B) demonstrated that the overall flower‐like morphology of the PAA@Cu^2+^ hNFs was retained after Hb adsorption. However, compared with the PAA@Cu^2+^ hNFs (Figure 2D), noticeable morphological changes were observed in the petal‐like nanosheets. The well‐defined and homogeneous interpetal voids characteristic of the nanoflowers became less distinct after Hb adsorption, while the petals appeared thicker, more compact, and partially merged (12C–I). These observations suggest that Hb molecules were successfully deposited onto the nanoflower surface, leading to partial surface coverage and structural rearrangement without disrupting the overall flower‐like architecture.

The EDX spectrum of the Hb‐loaded PAA@Cu^2+^ hNFs (Figure 12J) further supported successful protein immobilization. In addition to the characteristic C, O, P, and Cu signals observed for the PAA@Cu^2+^ hNFs, nitrogen (6.89 wt%) and sulfur (0.02 wt%) were clearly detected after Hb adsorption. Notably, a distinct iron signal, corresponding to the Fe Kα (∼6.40 keV) and Fe Kβ (∼7.06 keV) emission lines, was also detected (Figure 12J), whereas no iron signal was observed in the PAA@Cu^2+^ hNFs (Figures 5A, S4, and S5). Although present at low relative abundance (∼0.01 wt%), consistent with the small mass contribution of heme‐bound iron within the whole hemoglobin molecule (∼0.34% by mass) and its further dilution within the hybrid nanoflower matrix, this signal is chemically specific, since iron is exclusively associated with the heme group of Hb and is entirely absent from the PAA@Cu^2+^ hNF structure itself. Together with the nitrogen and sulfur signals, the detection of iron provides direct and specific evidence for successful Hb immobilization onto the hybrid nanoflowers. Furthermore, SEM‐EDX elemental mapping (Figure 12C–I) revealed a homogeneous distribution of C, O, P, Cu, N, S, and Fe throughout the nanoflower structure, indicating that Hb was uniformly distributed over the hybrid nanoflowers rather than being localized in specific regions. The hydrodynamic behavior of the hybrid nanoflowers also changed following Hb adsorption. The PAA@Cu^2+^ hNFs exhibited a maximum hydrodynamic diameter of 22.91 µm at pH 7 (Figure 6), whereas the Hb‐loaded nanoflowers showed a maximum diameter of approximately 6.5 µm under the same conditions (Figure 12K), indicating that Hb adsorption significantly altered the hydrodynamic properties of the hybrid nanoflowers. Moreover, the zeta potential of the Hb‐loaded PAA@Cu^2^^+^ hNFs gradually decreased from approximately +9 mV at pH 4 to about −20 mV at pH 9 (Figure 12L), demonstrating that the hybrid nanoflowers retained their pH‐responsive surface characteristics after Hb adsorption. Collectively, the SEM, EDX, elemental mapping, DLS, and zeta potential results provide complementary evidence for the successful adsorption of Hb onto PAA@Cu^2^^+^ hNFs and further support the proposed adsorption mechanism.

Until now, various materials including biomaterials, cryogels, beads, polymeric materials, and hybrid materials have been synthesized for protein adsorption. And also, the synthesis of new unique materials is increasing day by day. Nanoflowers have attracted significant attention in adsorption studies in recent years due to their superior characteristics, such as a high surface‐to‐volume ratio, high porosity, high stability, high adsorption capacity, selectivity, and reusability. In Table 1, a comparison is presented between synthesized PAA@Cu^2+^ hNFs and other studies reported in the literature for the separation of Hb.

**TABLE 1:** Comparison of synthesized PAA@Cu^2+^ hNFs with various materials for Hb separation.

Materials	Adsorption capacity	Ref.
Magnetic net‐poly(HEMA‐co‐AA) [net‐poly(2‐hydroxyethyl methacrylate‐co‐acrylic acid] microparticles	1263.5 mg/g	[56]
One‐dimensional (1D) magnetite hydroxyapatite nanorods on two‐dimensional (2D) reduced graphene oxide (MHAp/RGO)	1012 mg/g	[57]
Copper (II) affinity‐DTPA (Diethylene triamine pentacetate acid) functionalized magnetic composite	911.3 mg/g	[58]
The magnetic carbonized PDA@F127/ZIF‐67 hollow nanocages	834.3 mg/g	[59]
A supermacroporous poly(2‐hydroxyethyl methacrylate) (PHEMA)‐based Cu^2 +^‐attached bentonite particles embedded hybrid monolithic cryogel (Cu^2 +^‐ABPs EHMC)	521.6 mg/g	[51]
Molecularly imprinted polymer‐coated silicon nanowires	213.7 mg/g	[60]
Magnetic molecular imprinted nanoparticles (MMIPs)	169.29 mg/g	[61]
Amino‐functionalized silica surface molecularly imprinted polymers (MIPs@SiO2‐NH2)	90.3 mg/g	[62]
Our materials (PAA@Cu^2+^ hNFs)	961.98 mg/g	

### 3.7 Hb Desorption Study of PAA@Cu^2+^ hNFs and Pure Cu NFs

The pH and ionic strength of the medium are critical parameters in the desorption process. Generally, both parameters are used simultaneously in effective desorption studies [63]. In this study, the desorption yields of adsorbed Hb from PAA@Cu^2+^ hNFs and pure Cu NFs were measured in a 0.025 M acetate buffer solution, including 1 M NaCl at pH 5. The desorption yields of Hb for PAA@Cu^2+^ hNFs and pure Cu NFs were at about 96.1% and 95.8%, respectively. The electrostatic interaction between materials and Hb decreased under these conditions. Therefore, Hb is desorbed from the structure.

### 3.8 Reusability of PAA@Cu^2+^ hNFs hNFs and Pure Cu NFs

**FIGURE 13:**
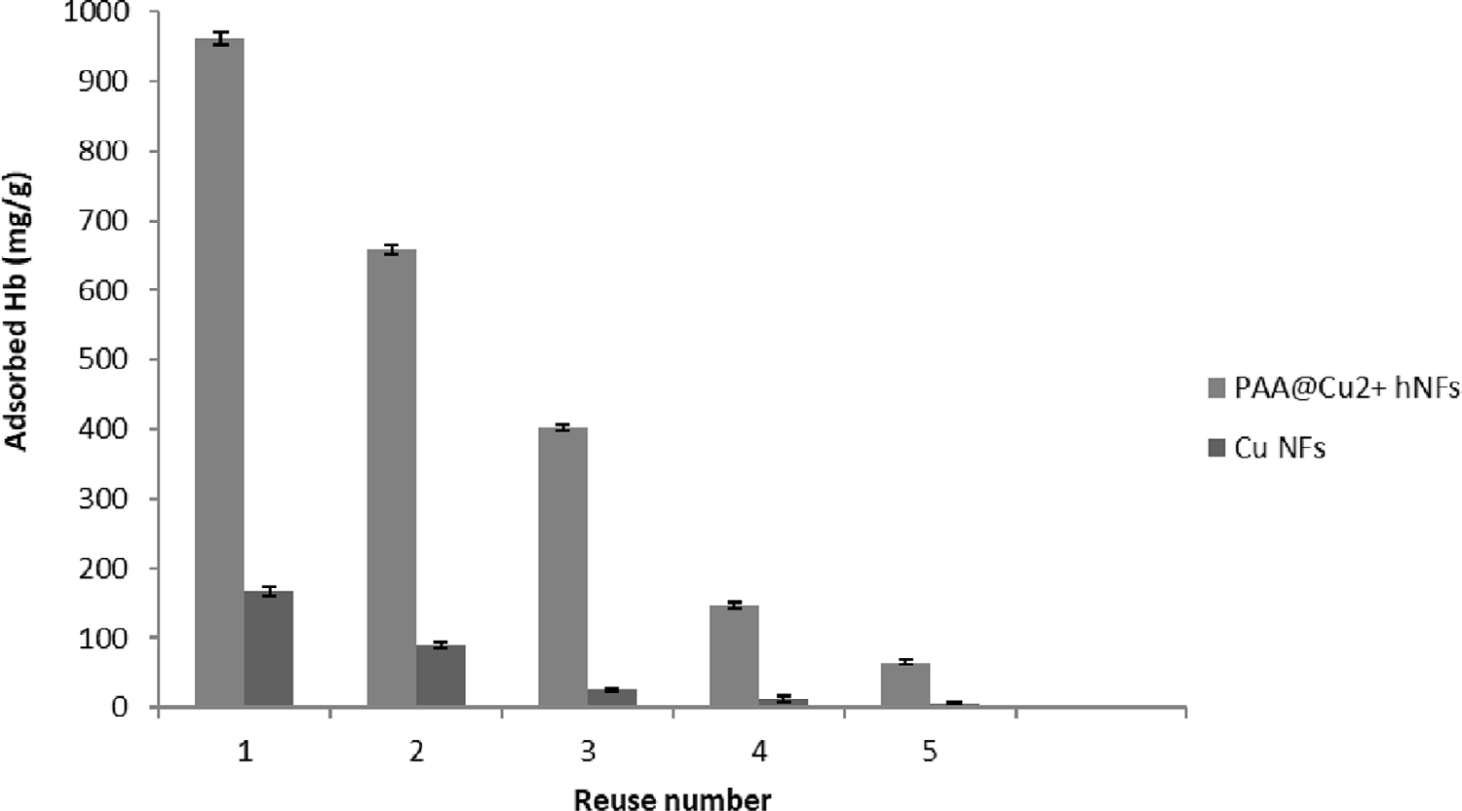
Reusability profiles of PAA@Cu^2+^ hNFs and pure Cu NFs (*n* = 3).

Reusability is an important aspect of the stability of materials [64]. Reusability profiles of PAA@Cu^2+^ hNFs and pure Cu NFs were given five cycles in Figure 13. As shown in Figure 13, PAA@Cu^2+^ hNFs demonstrated Hb adsorption capacity of 146.63 mg/g after four cycles. In brief, the adsorption capacity of the synthesized material was decreased by approximately 85%. Similarly, pure Cu NFs exhibited an Hb adsorption capacity of 26.52 mg/g after three cycles. In brief, the Hb adsorption capacity of this material was decreased by approximately 85%. In some studies, it is reported that this decrease in activity may be due to the scattering of the petals of the hybrid nanoflowers during the centrifugation process [65, 66].

## 4 Conclusions

In summary, for the first time, we fabricated the PAA@Cu^2+^ hNFs by using PAA and Cu^2+^ ions. Various characterization studies (SEM, SEM‐EDX, XRD, FTIR, Zeta potential, and DLS measurements) were performed to characterize the synthesized PAA@Cu^2+^ hNFs. The optimum morphology of PAA@Cu^2+^ hNFs was observed at pH 9 at a concentration of 0.02 mg mL^−1^ PAA and 0.8 mM Cu^2+^ ions. Synthesized PAA@Cu^2+^ hNFs at pH 9, compared to pure Cu NFs, morphologies at other pH levels are much more uniform, compact, and homogeneous in the areas between the petals. Various parameters, including pH, initial concentration, ionic strength, and temperature, influence adsorption conditions. For this reason, it is very important to investigate these parameters to determine the optimal conditions for adsorption. Based on this information, the effects of these parameters were systematically investigated in detail to determine the optimum adsorption conditions of Hb onto PAA@Cu^2+^ hNFs compared to pure Cu NFs. When examining the effect of pH on the adsorption capacities of PAA@Cu^2+^ hNFs and pure Cu NFs, it was observed that the adsorption capacity of PAA@Cu^2+^ hNF decreased below and above pH 7, but an increase was observed again at pH 4. This phenomenon was not observed for pure Cu NFs. The observed adsorption behavior is governed by the combined effects of both the physicochemical properties and the pH‐dependent characteristics of PAA@Cu^2^^+^ hNFs and Hb. This observed adsorption behavior can be explained by two main parameters. One of them is the isoelectric points of hNFs and Hb. The other is the stimuli responsive nature of PAA@Cu^2+^ hNFs. At pH 7, Hb adsorption capacities of PAA@Cu^2+^ hNFs were obtained to be 968.79 mg g^−1^ at 35°C after 24 h. The adsorption capacity of PAA@Cu^2+^ hNFs increased dramatically from 25°C to 35°C. This is due to the strong interactions between the carboxyl groups of PAA@Cu^2+^ hNFs and Hb. The desorption studies of Hb from PAA@Cu^2+^ hNFs and pure Cu NFs were performed in 0.025 M acetate buffer solution including 1 M NaCl at pH 5. The desorption yields of Hb for PAA@Cu^2+^ hNFs and pure Cu NFs were calculated as 96.1% and 95.8%, respectively. After desorption studies, the reusability of PAA@Cu^2+^ hNFs and pure Cu_3_(PO_4_)_2_.3H_2_O NFs for Hb adsorption was investigated through five cycles. After five cycles, PAA@Cu^2+^ hNFs and pure Cu NFs exhibited 64.91 and 6.17 mg/g of Hb adsorption capacity, respectively. The remarkably high adsorption capacity (968 mg g^−^^1^) suggests extensive interactions between hemoglobin molecules and the abundant functional groups available on the petal‐like surfaces of the PAA@Cu^2^^+^ hybrid nanoflowers. The structural characterization presented in Figure 12 further supports this interpretation by demonstrating surface‐related morphological changes following Hb adsorption. The combined characterization results suggest that hemoglobin adsorption mainly occurs through surface interactions without destroying the overall nanoflower framework, although localized rearrangement of the petal‐like structures is observed after adsorption.

According to the obtained results, the synthesized PAA@Cu^2+^ hNFs exhibited pH‐dependent adsorption/desorption behavior toward Hb and demonstrated high adsorption capacity, efficient desorption, and satisfactory reusability. These results suggest that PAA@Cu^2+^ hNFs are promising materials for Hb separation. The observed behavior is attributed to the combined effects of the pH‐responsive nature of the PAA‐containing hNFs and the pH‐dependent physicochemical properties of Hb. The combined SEM, FTIR, EDX, elemental mapping, DLS, and zeta potential analyses demonstrated that hemoglobin was successfully immobilized onto the PAA@Cu^2+^ hybrid nanoflowers while maintaining the overall flower‐like framework, providing additional insight into the adsorption mechanism.

The maximum adsorption capacity of 968 mg g^−^^1^ corresponds to the recovery of approximately 96.8% of hemoglobin from a 1 mg mL^−^^1^ Hb solution using only 3 mg of PAA@Cu^2+^ hNFs. This remarkably high adsorption efficiency highlights the excellent Hb‐scavenging capability of the developed hybrid nanoflowers and demonstrates their strong potential as an efficient hemoglobin adsorbent. We successfully demonstrated the high adsorption capacity, stability, and reusability of PAA@Cu^2+^ hNFs in practical Hb separation. Although the adsorption experiments were performed using purified hemoglobin solutions, the high adsorption capacity obtained in this study demonstrates the strong affinity of PAA@Cu^2+^ hybrid nanoflowers toward hemoglobin. Investigation of adsorption selectivity in more complex biological matrices will be the subject of future studies.

## Author Contributions

**Merve Durmus**: conceptualization, methodology, investigation, formal analysis, data curation, visualization, writing – original draft, writing – review and editing. **Nalan Özdemir**: conceptualization, methodology, investigation, supervision, writing – original draft, writing – review and editing. All authors have read and approved the final version of the manuscript.

## Funding

The author gratefully acknowledges the Council of Higher Education of Turkey (YÖK) for financial support through the 100/2000 PhD Scholarship Program.

## Conflicts of Interest

The authors declare no conflicts of interest.

## Supporting information




**Supporting File 1**: cbdv71762‐sup‐0001‐SuppMat.docx.

## Data Availability

The data supporting the findings of this study are available within the article and its Supporting Information.

## References

[1] J. Han , P. Luo , L. Wang , J. Wu , C. Li , and Y. Wang , “Construction of a Multienzymatic Cascade Reaction System of Coimmobilized Hybrid Nanoflowers for Efficient Conversion of Starch Into Gluconic Acid,” ACS Applied Materials & Interfaces 12, no. 13 (2020): 15023–15033, 10.1021/acsami.9b21511. 32156109

[2] H. Zhang , X. Fei , J. Tian , et al., “Synthesis and Continuous Catalytic Application of Alkaline Protease Nanoflowers–PVA Composite Hydrogel,” Catalysis Communications 116 (2018): 5–9, 10.1016/j.catcom.2018.07.015.

[3] S. V. Bhosale , M. Al Kobaisi , R. W. Jadhav , and L. A. Jones , “Flower‐Like Superstructures: Structural Features, Applications and Future Perspectives,” Chemical Record 21, no. 2 (2021): 257–283, 10.1002/tcr.202000129. 33215848

[4] J. Ge , J. Lei , and R. N. Zare , “Protein–Inorganic Hybrid Nanoflowers,” Nature Nanotechnology 7, no. 7 (2012): 428–432, 10.1038/nnano.2012.80. 22659609

[5] Y. Liu , X. Ji , and Z. He , “Organic–Inorganic Nanoflowers: From Design Strategy to Biomedical Applications,” Nanoscale 11, no. 37 (2019): 17179–17194, 10.1039/C9NR05446D. 31532431

[6] K. H. Kim , J. M. Jeong , S. J. Lee , B. G. Choi , and K. G. Lee , “Protein‐Directed Assembly of Cobalt Phosphate Hybrid Nanoflowers,” Journal of Colloid and Interface Science 484 (2016): 44–50, 10.1016/j.jcis.2016.08.059. 27585999

[7] D. Shcharbin , I. Halets‐Bui , V. Abashkin , et al., “Hybrid Metal‐Organic Nanoflowers and Their Application in Biotechnology and Medicine,” Colloids and Surfaces B: Biointerfaces 182 (2019): 110354, 10.1016/j.colsurfb.2019.110354. 31325775

[8] P. Liu , J. Fan , and X. Chu , “Immobilization of Arginase Using the Organic‐Inorganic Hybrid Nanoflower Strategy for L‐Ornithine Production,” Journal of Inorganic and Organometallic Polymers and Materials 35, no. 4 (2025): 2315–2326, 10.1007/s10904-024-03268-0.

[9] M. Zhang , Y. Zhang , C. Yang , C. Ma , and J. Tang , “Enzyme‐Inorganic Hybrid Nanoflowers: Classification, Synthesis, Functionalization and Potential Applications,” Chemical Engineering Journal 415 (2021): 129075, 10.1016/j.cej.2021.129075.

[10] S. Polepalli and C. Pulla Rao , “Protein Based Hybrid Materials of Metal Phosphate Nanoflowers and Gels for Water Remediation: Perspectives and Prospects,” Chemistry—An Asian Journal 20, no. 5 (2025): e202401352, 10.1002/asia.202401352. 39777918

[11] B. Bakar , M. Akbulut , F. Ulusal , A. Ulu , N. Özdemir , and B. Ateş , “Horseradish Peroxidase Immobilized Onto Mesoporous Magnetic Hybrid Nanoflowers for Enzymatic Decolorization of Textile Dyes: A Highly Robust Bioreactor and Boosted Enzyme Stability,” ACS Omega 9, no. 23 (2024): 24558–24573, 10.1021/acsomega.4c00703. PMC11170722 38882139

[12] L. Xu and S. Liu , “Rapid Cu(II)‐Directed Self Assembly of Esterified Tea Polyphenol Oligomers to Controlled Release Nanoflower Carrier,” Journal of Agricultural and Food Chemistry 69, no. 27 (2021): 7725–7732, 10.1021/acs.jafc.1c01425. 34189913

[13] H. Li , L. Cen , S. Liu , Z. Qiu , Y. Zhang , and X. Luo , “Preparation of Copper‐Based Protein‐Inorganic Hybrid Nanoflowers With Peroxidase‐Like Activity for Colorimetric Detection of Biothiols,” Analytica Chimica Acta 1377 (2025): 344685, 10.1016/j.aca.2025.344685. 41093535

[14] J. Lv , Y. Dong , Z. Gu , and D. Yang , “Programmable DNA Nanoflowers for Biosensing, Bioimaging, and Therapeutics,” Chemistry—A European Journal 26, no. 64 (2020): 14512–14524, 10.1002/chem.202002242. 32969061

[15] X. Li , Y. Xiong , M. Duan , et al., “Investigation on the Adsorption‐Interaction Mechanism of Pb(II) at Surface of Silk Fibroin Protein‐Derived Hybrid Nanoflower Adsorbent,” Materials 13, no. 5 (2020): 1241, 10.3390/ma13051241. PMC7085063 32182957

[16] A. Serinbaş , B. Önal , Ö. Acet , et al., “A New Application of Inorganic Sorbent for Biomolecules: IMAC Practice of Fe^3+^‐Nano Flowers for DNA Separation,” Materials Science and Engineering: C 113 (2020): 111020, 10.1016/j.msec.2020.111020. 32487418

[17] M. Turk , C. Altinkaynak , N. Yangin , and N. Özdemir , “Fabrication of Myoglobin Hybrid Nanoflowers for Decolorization Process of Evans Blue and congo Red,” Materials Letters 325 (2022): 132853, 10.1016/j.matlet.2022.132853.

[18] E. Yilmaz , I. Ocsoy , N. Ozdemir , and M. Soylak , “Bovine Serum Albumin‐Cu(II) Hybrid Nanoflowers: An Effective Adsorbent for Solid Phase Extraction and Slurry Sampling Flame Atomic Absorption Spectrometric Analysis of Cadmium and Lead in Water, Hair, Food and Cigarette Samples,” Analytica Chimica Acta 906 (2016): 110–117, 10.1016/j.aca.2015.12.001. 26772130

[19] A. H. Memon , R. Ding , Q. Yuan , Y. Wei , and H. Liang , “Facile Synthesis of Alcalase‐Inorganic Hybrid Nanoflowers Used for Soy Protein Isolate Hydrolysis to Improve Its Functional Properties,” Food Chemistry 289 (2019): 568–574, 10.1016/j.foodchem.2019.03.096. 30955650

[20] B. Sahu , S. Maity , A. Jain , and S. Banerjee , “Next‐Generation Stimuli‐Responsive Polymers for a Sustainable Tomorrow,” Chemical Communications 61, no. 66 (2025): 12265–12282, 10.1039/D5CC02729B. 40719016

[21] S. Tan , K. Saito , and M. T. Hearn , “Stimuli‐Responsive Polymeric Materials for Separation of Biomolecules,” Current Opinion in Biotechnology 53 (2018): 209–223, 10.1016/j.copbio.2018.02.011. 29549873

[22] Z. Zhou , M. Vázquez‐González , and I. Willner , “Stimuli‐Responsive Metal–Organic Framework Nanoparticles for Controlled Drug Delivery and Medical Applications,” Chemical Society Reviews 50, no. 7 (2021): 4541–4563, 10.1039/D0CS01030H. 33625421

[23] G. Roshan Deen , Y. L. Tan , M. R. Yalini , C. H. Mah , and T. W. Teo , “Synthesis, Swelling Characteristics, and Dye Adsorption Mechanism of a New Stimuli‐Responsive Cationic Hydrogel,” European Journal of Advanced Chemistry Research 3, no. 1 (2022): 12–24, 10.24018/ejchem.2022.3.1.86.

[24] A. M. Thomas , J. Peter , S. Nagappan , A. Mohan , and C. S. Ha , “Dual Stimuli‐Responsive Copper Nanoparticles Decorated SBA‐15: A Highly Efficient Catalyst for the Oxidation of Alcohols in Water,” Nanomaterials 10, no. 10 (2020): 2051, 10.3390/nano10102051. PMC7603010 33081325

[25] F. Gu and X. Ma , “Stimuli‐Responsive Polymers With Room‐Temperature Phosphorescence,” Chemistry—A European Journal 28, no. 15 (2022): e202104131, 10.1002/chem.202104131. 34882851

[26] T. Ramakrishnan , S. S. Kumar , S. J. S. Chelladurai , et al., “Recent Developments in Stimuli Responsive Smart Materials and Applications: An Overview,” Journal of Nanomaterials 2022 (2022): 4031059, 10.1155/2022/4031059.

[27] A. Fattah‐alhosseini , R. Chaharmahali , S. Alizad , M. Kaseem , and B. Dikici , “A Review of Smart Polymeric Materials: Recent Developments and Prospects for Medicine Applications,” Hybrid Advances 5 (2024): 100178, 10.1016/j.hybadv.2024.100178.

[28] B. Hosseini Monjezi , K. Kutonova , M. Tsotsalas , S. Henke , and A. Knebel , “Current Trends in Metal–Organic and Covalent Organic Framework Membrane Materials,” Angewandte Chemie International Edition 60, no. 28 (2021): 15153–15164, 10.1002/anie.202015790. PMC8359388 33332695

[29] M. Zhang , J. Qiao , and L. Qi , “Dual‐Functional Polymer‐Modified Magnetic Nanoparticles for Isolation of Lysozyme,” Analytica Chimica Acta 1035 (2018): 70–76, 10.1016/j.aca.2018.07.019. 30224146

[30] S. Liu , Z. Li , B. Yu , S. Wang , Y. Shen , and H. Cong , “Recent Advances on Protein Separation and Purification Methods,” Advances in Colloid and Interface Science 284 (2020): 102254, 10.1016/j.cis.2020.102254. 32942182

[31] B. Önal , Ö. Acet , V. Dzmitruk , et al., “First Protein Affinity Application of Cu^2+^‐Bound Pure Inorganic Nanoflowers,” Polymer Bulletin 79, no. 5 (2022): 3233–3251, 10.1007/s00289-021-03557-5.

[32] A. Kyrychenko , M. M. Blazhynska , M. V. Slavgorodska , and O. N. Kalugin , “Stimuli‐Responsive Adsorption of Poly(acrylic acid) Onto Silver Nanoparticles: Role of Polymer Chain Length and Degree of Ionization,” Journal of Molecular Liquids 276 (2019): 243–254, 10.1016/j.molliq.2018.11.130.

[33] H. Arkaban , M. Barani , M. R. Akbarizadeh , et al., “Polyacrylic Acid Nanoplatforms: Antimicrobial, Tissue Engineering, and Cancer Theranostic Applications,” Polymers 14, no. 6 (2022): 1259, 10.3390/polym14061259. PMC8948866 35335590

[34] D. Shao , K. Xu , X. Song , J. Hu , W. Yang , and C. Wang , “Effective Adsorption and Separation of Lysozyme With PAA‐Modified Fe_3_O_4_@Silica Core/Shell Microspheres,” Journal of Colloid and Interface Science 336, no. 2 (2009): 526–532, 10.1016/j.jcis.2009.02.061. 19464024

[35] X. Chen , S. Chen , and J. Wang , “A pH‐Responsive Poly(N‐isopropylacrylamide‐co‐acrylic acid) Hydrogel for the Selective Isolation of Hemoglobin From Human Blood,” Analyst 135, no. 7 (2010): 1736, 10.1039/C000465K. 20480063

[36] C. Müller , K. Leithner , S. Hauptstein , F. Hintzen , W. Salvenmoser , and A. Bernkop‐Schnürch , “Preparation and Characterization of Mucus‐Penetrating Papain/Poly(acrylic acid) Nanoparticles for Oral Drug Delivery Applications,” Journal of Nanoparticle Research 15, no. 1 (2012): 1353, 10.1007/s11051-012-1353-z.

[37] A. R. Mahdavian and M. A. S. Mirrahimi , “Efficient Separation of Heavy Metal Cations by Anchoring Polyacrylic Acid on Superparamagnetic Magnetite Nanoparticles Through Surface Modification,” Chemical Engineering Journal 159, no. 1 (2010): 264–271, 10.1016/j.cej.2010.02.041.

[38] V. A. Tran and S. W. Lee , “pH‐Triggered Degradation and Release of Doxorubicin From Zeolitic Imidazolate Framework‐8 (ZIF8) Decorated With Polyacrylic Acid,” RSC Advances 11, no. 16 (2021): 9222–9234, 10.1039/D0RA10423J. PMC8695245 35423461

[39] M. Bakhshpour and A. Denizli , “Preparation of Bacterial Cellulose/Vinyl Imidazole‐Based Membranes for Selective Purification of Hemoglobin,” Hacettepe Journal of Biology and Chemistry 49, no. 4 (2021): 423–431, 10.15671/hjbc.921540.

[40] C. Gulmez , C. Altinkaynak , M. Turk , N. Ozdemir , and O. Atakisi , “Hemoglobin‐Inorganic Hybrid Nanoflowers With Different Metal Ions as Potential Oxygen Carrying Systems,” Chemistry & Biodiversity 19 (2022): e202100683, 10.1002/cbdv.202100683. 34813152

[41] S. Khanlari and M. A. Dubé , “Effect of pH on Poly(acrylic acid) Solution Polymerization,” Journal of Macromolecular Science, Part A 52, no. 8 (2015): 587–592, 10.1080/10601325.2015.1050628.

[42] N. Özdemir , C. Altinkaynak , M. Türk , F. Geçili , and S. Tavlaşoğlu , “Amino Acid‐Metal Phosphate Hybrid Nanoflowers (AaHNFs): Their Preparation, Characterization and Anti‐Oxidant Capacities,” Polymer Bulletin 79 (2022): 9697–9716, 10.1007/s00289-021-03973-7.

[43] M. M. Bradford , “A Rapid and Sensitive Method for the Quantitation of Microgram Quantities of Protein Utilizing the Principle of Protein‐Dye Binding,” Analytical Biochemistry 72, no. 1 (1976): 248–254, 10.1016/0003-2697(76)90527-3. 942051

[44] Z. Lin , Y. Xiao , Y. Yin , W. Hu , W. Liu , and H. Yang , “Facile Synthesis of Enzyme‐Inorganic Hybrid Nanoflowers and Its Application as a Colorimetric Platform for Visual Detection of Hydrogen Peroxide and Phenol,” ACS Applied Materials & Interfaces 6, no. 13 (2014): 10775–10782, 10.1021/am502757e. 24937087

[45] P. Lertsarawut , K. Hemvichian , T. Rattanawongwiboon , and P. Suwanmala , “Dye Adsorbent Prepared by Radiation‐Induced Graft Polymerization of Acrylic Acid Onto Carboxymethyl Cellulose,” Journal of Physics: Conference Series 1285 (2019): 012023, 10.1088/1742-6596/1285/1/012023.

[46] T. Huang , Z. Su , K. Hou , et al., “Advanced Stimuli‐Responsive Membranes for Smart Separation,” Chemical Society Reviews 52, no. 13 (2023): 4173–4207, 10.1039/D2CS00911K. 37184537

[47] S. Devineau , K.‐I. Inoue , R. Kusaka , et al., “Change of the Isoelectric Point of Hemoglobin at the Air/Water Interface Probed by the Orientational Flip‐Flop of Water Molecules,” Physical Chemistry Chemical Physics 19, no. 16 (2017): 10292–10300, 10.1039/C6CP08854F. 28383588

[48] H. F. Bunn and B. G. Forget , Hemoglobin: Molecular, Genetic and Clinical Aspects (W. B. Saunders Company, Philadelphia, PA, 1986) ISBN 0‐7216‐2181‐3.

[49] M. Wiśniewska , T. Urban , E. Grządka , V. I. Zarko , and V. M. Gun'ko , “Comparison of Adsorption Affinity of Polyacrylic Acid for Surfaces of Mixed Silica–Alumina,” Colloid and Polymer Science 292, no. 3 (2014): 699–705, 10.1007/s00396-013-3103-x. PMC3940901 24610970

[50] S. V. Kazakov , V. I. Muronetz , M. B. Dainiak , V. A. Izumrudov , I. Y. Galaev , and B. Mattiasson , “Light Scattering Study of the Antibody‐Poly(methacrylic acid) and Antibody‐Poly(acrylic acid) Conjugates in Aqueous Solutions,” Macromolecular Bioscience 1, no. 4 (2001): 157–163, 10.1002/1616-5195(20010601)1:4<157::AID-MABI157>3.0.CO;2-S.

[51] N. Y. Baran , Ö. Acet , and M. Odabaşı , “Efficient Adsorption of Hemoglobin From Aqueous Solutions by Hybrid Monolithic Cryogel Column,” Materials Science and Engineering: C 73 (2017): 15–20, 10.1016/j.msec.2016.12.036. 28183592

[52] R. Blanco , A. Arai , N. Grinberg , M. Yarmush , and B. L. Karger , “Role of Association on Protein Adsorption Isotherms,” Journal of Chromatography A 482, no. 1 (1989): 1–12, 10.1016/S0021-9673(01)93202-9. 2613775

[53] K. D. Collins and M. W. Washabaugh , “The Hofmeister Effect and The Behaviour of Water at Interfaces,” Quarterly Reviews of Biophysics 18, no. 4 (1985): 323–422, 10.1017/S0033583500005369. 3916340

[54] M. Hassan , E. Azzazy , and R. H. Christenson , “All About Albumin: Biochemistry, Genetics, and Medical Applications. Theodore Peters, Jr. San Diego, CA: Academic Press, 1996, 432 pp, $85.00. ISBN 0‐12‐552110‐3,” Clinical Chemistry 43 (1997): 2014a–2015, https://academic.oup.com/clinchem/article/43/10/2014a/5640631.

[55] M. Wiśniewska and S. Chibowski , “Influence of Temperature and Purity of Polyacrylic Acid on Its Adsorption and Surface Structures at the ZrO_2_/Polymer Solution Interface,” Adsorption Science & Technology 23, no. 8 (2005): 655–667, 10.1260/026361705775373279.

[56] K. Erol and K. Köse , “Efficient Polymeric Material for Separation of Human Hemoglobin,” Artificial Cells, Nanomedicine, and Biotechnology 45, no. 1 (2017): 39–45, 10.1080/21691401.2016.1233112. 27662700

[57] G. Bharath , K. Rambabu , A. Hai , S. Anwer , F. Banat , and N. Ponpandian , “Synthesis of One‐Dimensional Magnetite Hydroxyapatite Nanorods on Reduced Graphene Oxide Sheets for Selective Separation and Controlled Delivery of Hemoglobin,” Applied Surface Science 501 (2020): 144215, 10.1016/j.apsusc.2019.144215.

[58] J. Wang , H. Guan , Q. Liang , and M. Ding , “Construction of Copper(II) Affinity‐ DTPA Functionalized Magnetic Composite for Efficient Adsorption and Specific Separation of Bovine Hemoglobin From Bovine Serum,” Composites Part B: Engineering 198 (2020): 108248, 10.1016/j.compositesb.2020.108248.

[59] S. Tan , Y. Long , Q. Han , H. Guan , Q. Liang , and M. Ding , “Designed Fabrication of Polymer‐Mediated MOF‐Derived Magnetic Hollow Carbon Nanocages for Specific Isolation of Bovine Hemoglobin,” ACS Biomaterials Science & Engineering 6, no. 3 (2020): 1387–1396, 10.1021/acsbiomaterials.9b01793. 33455361

[60] T. Chen , M. Shao , H. Xu , S. Zhuo , S. Liu , and S. T. Lee , “Molecularly Imprinted Polymer‐Coated Silicon Nanowires for Protein Specific Recognition and Fast Separation,” Journal of Materials Chemistry 22, no. 9 (2012): 3990, 10.1039/C2JM14329A.

[61] Y. Su , B. Qiu , C. Chang , et al., “Separation of Bovine Hemoglobin Using Novel Magnetic Molecular Imprinted Nanoparticles,” RSC Advances 8, no. 11 (2018): 6192–6199, 10.1039/C7RA12457K. PMC9078349 35539629

[62] Z. Zhang and L. Li , “Efficient Synthesis of Molecularly Imprinted Polymers With Bio‐Recognition Sites for the Selective Separation of Bovine Hemoglobin,” Journal of Separation Science 41, no. 11 (2018): 2479–2487, 10.1002/jssc.201701479. 29466619

[63] A. Heebøll‐Nielsen , S. F. L. Justesen , and O. R. T. Thomas , “Fractionation of Whey Proteins With High‐Capacity Superparamagnetic Ion‐Exchangers,” Journal of Biotechnology 113, no. 1 (2004): 247–262, 10.1016/j.jbiotec.2004.06.008. 15380659

[64] X. Liang , Y. Liu , K. Wen , W. Jiang , and Q. Li , “Immobilized Enzymes in Inorganic Hybrid Nanoflowers for Biocatalytic and Biosensing Applications,” Journal of Materials Chemistry B 9, no. 37 (2021): 7597–7607, 10.1039/D1TB01476E. 34596205

[65] H. R. Lee , M. Chung , M. I. Kim , and S. H. Ha , “Preparation of Glutaraldehyde‐treated Lipase‐Inorganic Hybrid Nanoflowers and Their Catalytic Performance as Immobilized Enzymes,” Enzyme and Microbial Technology 105 (2017): 24–29, 10.1016/j.enzmictec.2017.06.006. 28756857

[66] S. S. Maurya , S. S. Nadar , and V. K. Rathod , “Dual Activity of Laccase‐Lysine Hybrid Organic–Inorganic Nanoflowers for Dye Decolourization,” Environmental Technology & Innovation 19 (2020): 100798, 10.1016/j.eti.2020.100798.

