# Supplementary material for: Hemoglobin Adsorption/Desorption Studies of New Generation Stimuli Responsive Polyacrylic Acid‐Cu2+ Hybrid Nanoflowers (PAA@Cu2+ hNFs)
Source: Chem Biodivers. 2026 Sep 27;23(9):e71762. doi: 10.1002/cbdv.71762 (PMC13616474; doi:10.1002/cbdv.71762)
Supplement: Supplementary file 1 [file CBDV-23-e71762-s001.docx]

**Figure S1**. BSA Calibration curve

**Figure S2.** Diameter measurements of PAA@Cu^2+^ hNFs synthesized at different pHs using Image J (A) pH 6 (B) pH 7 (C) pH 8 (D) pH 9

**Figure S3.** Diameter measurements of Cu NFs synthesized at different pHs using Image J (A) pH 6 (B) pH 7 (C) pH 8 (D) pH 9

**Table S1.** Pore size, total pore volume and surface area of the synthesized PAA@Cu^2+^ hNFs and Cu NFs structures

|  | **Pore Size (nm)** | **Total Pore Volume × 10^-3^ (cm^3^/g)** | **Surface Area (m^2^/g)** |
| --- | --- | --- | --- |
| **PAA@Cu^2+^ hNFs (pH 7)** | 29.82 | 241.90 | 71.14 |
| **PAA@Cu^2+^ hNFs (pH 8)** | 21.92 | 295.16 | 82.26 |
| **PAA@Cu^2+^ hNFs (pH 9)** | 19.30 | 450.78 | 115.14 |
| **Cu NFs** | 15.7181 | 132.185 | 44,2666 |

**Figure S4.** Elemental mapping for the synthesized PAA@Cu^2+^ hNFs (A) SEM image, (B) all-in-one SEM-EDX elemental mapping image of C, O, P and Cu elements, (C) C, (D) O, (E) P (F) Cu

**Figure S5.** Elemental mapping for synthesized Cu NFs (A) SEM image, (B) O, (C) P, (D) Cu (E) EDX results of the Cu NFs

**Figure S6**. XRD patterns of Cu NFs. The peak position of the Cu_3_(PO_4_)_2_. 3H_2_O (JCPDS 00—22-0548) located for comparison

**Figure S7**. Zeta potential measurement of PAA@Cu^2+^ hNFs synthesized at pH 9 (A) pH 5 (B) pH 6 (C) pH 7 (D) pH 8 (F) pH 9

**Figure S8.** Zeta potential measurement of Cu NFs synthesized at pH 7 (A) pH 5 (B) pH 6 (C) pH 7 (D) pH 8 (F) pH 9
